# A synergistic enhancement of the Ivy algorithm for GAN-based imbalanced classification

**DOI:** 10.1038/s41598-025-32510-z

**Published:** 2025-12-21

**Authors:** Hanjie Xu, Jian Xiong, Jinyu Wu, Xianlai Zhou, Ronghu Xu, Haozheng Wu

**Affiliations:** 1 Guangzhou Xinhua University, Guangzhou, 510520 China; 2 Guangdong Polytechnic Normal University, Guangzhou, 510665 China

**Keywords:** Swarm intelligence, Ivy algorithm, Multi-strategy synergistic enhancement, Hyperparameter optimization, Imbalanced data classification, Engineering, Mathematics and computing

## Abstract

The Ivy Algorithm (IVYA), a swarm intelligence algorithm inspired by plant growth, presents a novel framework for optimization. To unlock its full potential in complex, high-dimensional problems, it is crucial to address the fundamental challenge of balancing exploration and exploitation, which can impact overall search efficiency and solution quality. To this end, this paper proposes an Enhanced Ivy Algorithm (E-IVYA) that integrates three synergistic mechanisms. First, a dynamic perturbation framework combining symmetric and asymmetric exploration is introduced to maintain population diversity. Second, a dynamic escape mechanism based on elite differential mutation is employed to prevent search stagnation and effectively escape from local optima. Third, an adaptive movement strategy inspired by the Sine-Cosine Algorithm is integrated to achieve a more adaptive balance between global exploration and local exploitation. The performance of the proposed E-IVYA was rigorously evaluated through two distinct phases. Initially, its optimization capabilities were benchmarked against a wide range of classic and advanced algorithms on the challenging IEEE CEC 2014 and 2017 test suites. Subsequently, its practical utility was validated by applying it to the complex task of automating the hyperparameter optimization of Generative Adversarial Networks (GANs) for imbalanced data classification. The experimental results demonstrate E-IVYA’s superior performance. On the standard benchmarks, E-IVYA consistently ranked as a top-performing algorithm. In the practical application, the E-IVYA-optimized GAN model achieved a minority class F1-Score of 0.87 on the highly imbalanced Credit-Card Fraud dataset, significantly outperforming models augmented with standard techniques like SMOTE (0.71). These findings confirm that E-IVYA is a robust and efficient tool for tackling complex optimization problems, particularly in the domain of automated machine learning.

## Introduction

In many real-world machine learning applications, the Class Imbalance Problem (CIP) is a pervasive challenge that can severely impact the performance of traditional classification models ^1–3^. This issue, where samples of some classes vastly outnumber others, is particularly critical in domains such as medical diagnosis and financial fraud detection, where the misclassification of minority class instances carries significant consequences ^4,5^. While various data-level approaches exist, classic oversampling techniques like SMOTE, although widely used, may generate synthetic samples that are noisy, borderline, or insufficiently diverse, thus failing to capture the true underlying data distribution ^5^. To overcome these limitations, data augmentation via Generative Adversarial Networks (GANs) has emerged as a promising approach, capable of generating high-quality, realistic synthetic samples for the minority class, thereby balancing the dataset and improving classifier performance ^6,7^. However, the success of GANs is critically dependent on an optimal set of hyperparameters, and their training is notoriously sensitive and complex ^8,9^. Manually tuning these parameters is often inefficient and computationally prohibitive, turning the task of GAN configuration into a complex, high-dimensional optimization problem in its own right ^10^. This challenge falls squarely into the domain of optimization, which is a central task in scientific inquiry and engineering design ^11–13^. Due to the complexity and high computational demands, metaheuristic algorithms have emerged as a class of effective approximate methods for solving such problems ^14,15^.

Metaheuristic algorithms simulate natural phenomena, physical laws, or social behaviors to intelligently search the solution space ^16^. Based on their source of inspiration, these methods are broadly classified into several major branches, including Evolutionary Algorithms (EAs) like the Genetic Algorithm (GA) ^17^, and Swarm Intelligence (SI) algorithms like Particle Swarm Optimization (PSO) ^18^. The field continues to evolve rapidly, with numerous high-performing algorithms proposed recently, such as the Starling Murmurination Optimizer (SMO) ^19^ and the White Shark Optimizer (WSO) ^20^. However, it is important to acknowledge that this rapid proliferation has sparked a critical discussion within the scientific community regarding the novelty of “metaphor-based” algorithms ^21,22^. We concur that an algorithm’s value is determined not by its narrative, but by the principled design of its search operators and its empirical performance. Our focus, therefore, is on principled algorithm engineering.

Despite the wide variety of metaheuristic algorithms, nearly all face a core challenge: achieving an effective balance between global exploration and local exploitation ^23^. Exploration allows the algorithm to search the entire solution space broadly, while exploitation involves a fine-grained search within promising regions to refine solution precision ^24,25^. For an intricate task such as GAN hyperparameter optimization, an overemphasis on exploitation can trap the search in a suboptimal set of parameters, whereas excessive exploration may lead to inefficient convergence ^26,27^. This underscores the necessity of developing improved algorithms specifically engineered to navigate this trade-off more intelligently.

In line with the algorithm engineering focus established above, this paper treats the recently introduced Ivy Algorithm (IVYA) ^28^ not as a novel metaphor, but as a representative baseline structure that exhibits these exact challenges. To this end, we propose an Enhanced Ivy Algorithm (E-IVYA). We introduce a synergistic set of non-metaphorical enhancement strategies, designed to directly address the fundamental, general challenges of metaheuristic optimization:

1. *To counteract the loss of population diversity*, we introduce a Dynamic Perturbation Framework.
2. *To overcome search stagnation* in local optima, we design a Dynamic Perturbation Escape Mechanism.
3. *To resolve the static balance between search behaviors*, we incorporate an Adaptive Movement and Balancing Mechanism.

By synergistically combining these improvements, this work aims to develop a robust and efficient optimization tool, validated through rigorous benchmarking and a complex, real-world application in automated machine learning.

### Motivation and proposed enhancements for the Ivy algorithm

Within the vast landscape of metaheuristic algorithms, the selection of a suitable foundational algorithm for in-depth research is of paramount importance. In line with the principled algorithm engineering focus established in our Introduction, this study selects the Ivy Algorithm (IVYA) ^28^ as the basis for enhancement.

Our decision is predicated not on its bio-inspired narrative, but rather on its clear and modular structure, which provides an ideal ’experimental platform’. This platform allows us to rigorously test the efficacy of several promising enhancement strategies designed to overcome common challenges in the field of metaheuristics.

Our preliminary analysis confirmed that the baseline IVYA framework, like many population-based algorithms, exhibits distinct and addressable limitations, particularly in maintaining population diversity and handling search stagnation. Therefore, enhancing IVYA provides a valuable case study for exploring the design paradigms of robust optimization algorithms by systematically addressing these general, non-metaphorical deficiencies. To this end, this paper proposes an Enhanced Ivy Algorithm (E-IVYA), which integrates three synergistic strategies. The primary motivations and corresponding engineering contributions–each targeting a specific, well-defined limitation–are summarized as follows:

1. *A Dynamic Perturbation Framework Combining Symmetric and Asymmetric Exploration:* To address the common challenge of maintaining population diversity, a phenomenon that can lead to premature convergence in many metaheuristics, E-IVYA introduces a novel framework that functions as a hierarchical diversity control system. Its foundational component, a symmetric exploration operator based on elite-guided oppositional learning, continuously refines the population structure. This is complemented by a macro-level intervention, an asymmetric shock operator based on an adaptive t-distribution, which is probabilistically triggered during stagnation to introduce radical, non-patterned perturbations, thereby ensuring the population maintains its exploratory potential.
2. *Dynamic Perturbation Escape Mechanism based on Elite Differential Mutation:* To equip the algorithm with a dedicated mechanism for addressing search stagnation, where individuals can become trapped in local optima, we have designed this mechanism. It employs a stagnation counter to detect periods of non-improvement. When stagnation is identified, it triggers an intelligent perturbation derived from the differential vectors of elite individuals in the current population. This creates a guided, high-potential leap away from the local trap, effectively reactivating the search process by leveraging the collective intelligence of the best-found solutions.
3. *An Adaptive Movement and Balancing Mechanism Based on Trigonometric Functions:* To achieve a more adaptive and dynamic balance between search behaviors according to the evolving requirements of the optimization process, E-IVYA incorporates this mechanism. It integrates the periodic oscillating properties of sine and cosine functions into the position update formula. Coupled with a parameter that decays dynamically with iterations, this mechanism enables a seamless transition from large-scale, exploratory movements in the early phases to small-scale, precise adjustments in the later stages, thus optimizing the exploration-exploitation trade-off.

By synergistically combining these three targeted improvements, the E-IVYA framework demonstrates significant enhancements in robustness, convergence accuracy, and overall optimization efficiency. This is achieved by *systematically engineering solutions to address the primary, well-defined limitations of the baseline algorithm.*

## Related work

### A brief introduction to the Ivy algorithm

The Ivy Algorithm (IVYA) is a novel nature-inspired metaheuristic optimization algorithm ^28^. It draws inspiration from the intelligent behaviors of ivy plants, such as their ability to grow, climb, and intertwine towards sunlight. IVYA aims to provide effective solutions for complex global optimization problems. The algorithm simulates key mechanisms of an ivy population, including initialization, growth strategies, and selection. The main steps of IVYA are as follows:

*Step 1* Initialize the Ivy Population and Growth Vectors

Initially, a population of $N_{Pop}$ ivy plants is created. Each plant $I_i$ represents a potential solution. The position of each plant is randomly initialized within the search space defined by lower bounds $I_{min}$ and upper bounds $I_{max}$. Equation (1) shows this initialization:

1 $$\begin{aligned} I_{i} = I_{min} + \text {rand}(1,D) \odot (I_{max} - I_{min}) \end{aligned}$$

where $I_{i}$ is the position vector of the *i*-th plant, *D* is the problem’s dimension, and $\text {rand}(1,D)$ is a vector of *D* uniformly distributed random numbers in [0, 1]. The symbol $\odot$ denotes the Hadamard product.

Each plant $I_i$ also possesses a Growth Vector (GV), $\Delta Gv_i$. For the first iteration (Iter=1), the initial GV is calculated using Eq. (2):

2 $$\begin{aligned} \Delta Gv_{i} = I_{i} \oslash (I_{max} - I_{min}) \end{aligned}$$

where $\oslash$ denotes the Hadamard division.

*Step 2* Ivy Growth and Movement Strategy

In each subsequent iteration, ivy plants update their GVs and generate new positions. This process involves several sub-steps:

*Sub-step 2.1. Update Existing Growth Vector*:

The GV, $\Delta Gv_i$, of the current plant $I_i$ is updated using Eq. (3):

3 $$\begin{aligned} \Delta Gv_{i}(t+1) = \text {rand}^2 \cdot (N(1,D) \odot \Delta Gv_{i}(t)) \end{aligned}$$

where $\Delta Gv_{i}(t)$ is the GV at iteration *t*, $\text {rand}$ is a scalar random number in [0, 1], and *N*(1, *D*) is a vector of *D* random numbers from the standard normal distribution.

*Sub-step 2.2. Generate New Position*:

A new position $I_{i}^{\text {new}}$ is generated based on a decision parameter $\beta = (2 + \text {rand})/2$ and a comparison between the fitness $f(I_i)$ of the current plant and the fitness $f(I_{Best})$ of the best plant in the population.

- If $f(I_i) < \beta \cdot f(I_{Best})$, the plant employs a local search strategy (climbing growth). The new position is generated by learning from a ’more vital neighbor’ $I_{ii}$ (selected based on specific rules) as shown in Eq. (4):
  4 $$\begin{aligned} I_{i}^{\text {new}} = I_{i} + |N(1,D)| \odot (I_{ii} - I_{i}) + N(1,D) \odot \Delta Gv_{i} \end{aligned}$$
  where |*N*(1, *D*)| contains the absolute values of elements in *N*(1, *D*).
- Otherwise, the plant employs a global search strategy (spreading towards light). The new position is primarily guided by the best plant $I_{Best}$, as shown in Eq. (5):
  5 $$\begin{aligned} I_{i}^{\text {new}} = I_{Best} \odot (\text {rand}(1,D) + N(1,D) \odot \Delta Gv_{i}) \end{aligned}$$

*Sub-step 2.3. Update Growth Vector for the New Position*:

After generating $I_{i}^{\text {new}}$, its corresponding GV, $\Delta Gv_{i}^{\text {new}}$, is updated using Eq. (6):

6 $$\begin{aligned} \Delta Gv_{i}^{\text {new}} = I_{i}^{\text {new}} \oslash (I_{max} - I_{min}) \end{aligned}$$

*Sub-step 2.4. Boundary Handling*:

All newly generated positions $I_{i}^{\text {new}}$ are checked and adjusted to ensure they remain within the predefined search space boundaries $[I_{min}, I_{max}]$.

*Step 3* Population Update and Selection Mechanism

At the end of each iteration, the population from the previous iteration is merged with the newly generated population of ivy plants. This combined population is then sorted based on the fitness values of all individual plants, from best to worst. To maintain a constant population size, the top $N_{Pop}$ plants with the best fitness values are selected to form the population for the next iteration. This elitist selection ensures that promising solutions are preserved and carried forward.

IVYA continues these iterative steps, simulating the intelligent growth behavior of ivy, and balancing exploration with exploitation to converge towards the global optimal solution.

### An overview of SCA

The Sine-Cosine Algorithm (SCA) is a metaheuristic algorithm designed to solve optimization problems, particularly those with unknown search spaces ^29^. SCA utilizes the mathematical properties of sine and cosine functions to iteratively update the position of each search agent in the solution space, aiming to find the optimal solution ^30,31^. For a given problem, the set of variables, constraints, and the objective function together define its search space ^32^. This search space may contain multiple local optima, but only one is the global optimum. SCA balances exploration and exploitation to identify promising regions within the search space and ultimately converge to the global optimum ^33^.

The operational mechanism of SCA involves different phases. During the initial exploration phase, SCA combines random solutions and incorporates a high degree of randomness in the movement of search agents. This encourages a broad search to locate promising areas. As the algorithm transitions to the development (exploitation) phase, the changes in solution positions become more gradual. The stochastic variation in this later stage is significantly smaller than in the exploration phase. The update of the optimal solution employs Eqs. (1) or (2) based on the condition of parameter $r_4^t$.

The core of SCA is its position update mechanism, which dictates how each search agent moves towards the best solution found so far. The process of updating an agent $X_i^t$ to its new position $X_i^{t+1}$ is guided by the current best solution $P_i^t$ (the destination point). This update selectively uses either sine or cosine functions, based on the value of a random parameter $r_4^t$. The position update equations are as follows:

7 $$\begin{aligned} X_i^{t+1}= & X_i^t + r_1^t \times \sin (r_2^t) \times |r_3^t P_i^t - X_i^t|, \quad \text {if } r_4^t < 0.5 \end{aligned}$$

8 $$\begin{aligned} X_i^{t+1}= & X_i^t + r_1^t \times \cos (r_2^t) \times |r_3^t P_i^t - X_i^t|, \quad \text {if } r_4^t \ge 0.5 \end{aligned}$$

In these equations, $X_i^t$ is the position of the current solution *i* at iteration *t*, and $P_i^t$ is the position of the best solution found up to iteration *t*. The parameters $r_1^t, r_2^t, r_3^t,$ and $r_4^t$ are crucial for guiding the search. Parameter $r_1^t$ determines the region of the next position (or the direction of movement); its value is typically adjusted over iterations to shift from exploration to exploitation. Parameter $r_2^t$ defines the distance the movement should cover towards or away from the target. Parameter $r_3^t$ assigns a random weight to the destination, stochastically emphasizing (if $r_3^t > 1$) or de-emphasizing (if $r_3^t < 1$) the destination’s influence on the movement distance. Finally, $r_4^t$ is a uniformly distributed random number used to switch between the sine and cosine functions. Through the iterative application of these updates and the adaptive nature of its parameters, SCA navigates the search space to find optimal or near-optimal solutions.

### Advanced metaheuristic strategies and applications in deep learning

The continuous evolution of the metaheuristic field has led to several key research paradigms aimed at enhancing optimization performance. A prominent research direction focuses on improving a single base algorithm by integrating multiple enhancement mechanisms to systematically address inherent limitations such as premature convergence. For example, Wang et al. ^34^ proposed an improved reptile search algorithm with multi-population evolution for image segmentation, while Jia et al. ^35^ developed a multi-strategy enhanced dung beetle optimizer for engineering design. These studies validate the effectiveness of synergistically combining strategies within a single framework. Our work on E-IVYA follows a similar philosophy, focusing on the internal enhancement of the base IVYA to create a more robust and powerful optimizer.

Another powerful paradigm is the creation of hybrid metaheuristic frameworks, which combine the operators or strategies of two or more distinct algorithms, often to leverage the exploration strengths of one with the exploitation prowess of another. For instance, recent research has explored *the FOX-TSA hybridization* ^36^, *FOXTSA and Foxtsage-type optimizers* ^37,38^, *which integrate metaheuristic and learning-based refinement mechanisms*. In contrast to these high-level hybridization strategies, our proposed E-IVYA focuses on the deep, synergistic integration of multiple mechanisms within a single algorithmic core, allowing for a more seamless and adaptive control over the search process.

The application of these advanced metaheuristics to automate the tuning of deep learning models, particularly Generative Adversarial Networks (GANs), is a challenging and highly relevant research frontier. Due to the high-dimensional and computationally expensive nature of the hyperparameter search space, metaheuristics offer a significant advantage over manual tuning. Recent studies have demonstrated this potential: an evolutionary approach was used for hyperparameter tuning of a GAN for melanoma detection ^39^, and a Variable Neighborhood Search (VNS) was applied to a GAN-based seismic interpolator ^40^. Beyond just tuning, metaheuristics like Particle Swarm Optimization (PSO) have been used for GAN architecture search ^41^ and to optimize ANN models ^42^. These works confirm the viability of applying metaheuristics to complex neural network optimization tasks. Our research contributes to this domain by applying a novel, multi-strategy enhanced algorithm (E-IVYA) to the GAN hyperparameter optimization problem, demonstrating its practical utility in solving computationally expensive, real-world automated machine learning challenges.

## Methods

### A dynamic perturbation framework combining symmetric and asymmetric exploration

**Fig. 1:**
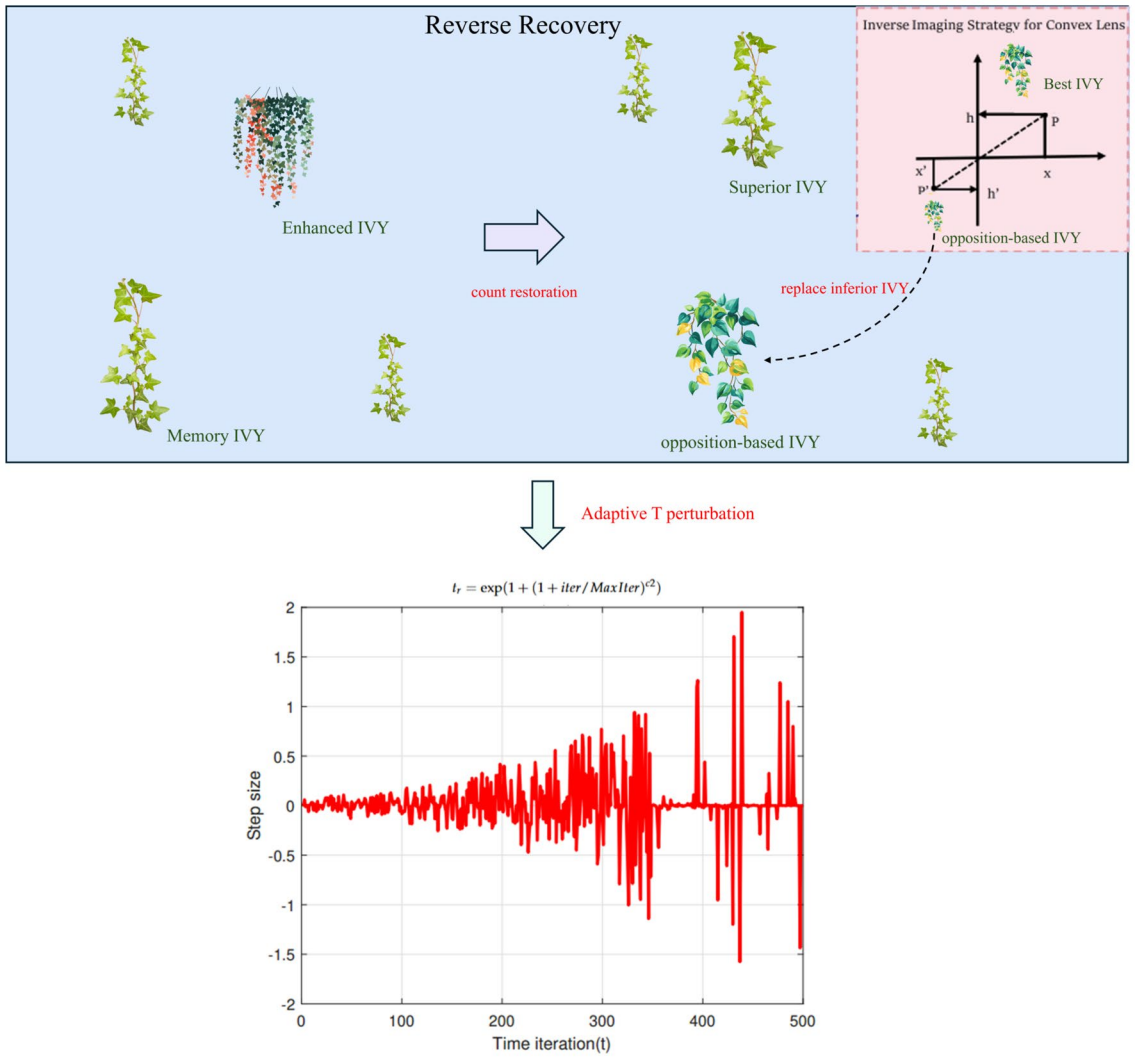
A Dynamic Perturbation Framework Combining Symmetric and Asymmetric Exploration.

To systematically address the decay of population diversity that can lead to premature convergence, this paper introduces a novel dynamic perturbation framework, conceptually illustrated in Fig.  1. This framework integrates two mechanistically distinct yet complementary operators to form a resilient diversity maintenance system.

The foundational component is a *symmetric exploration operator*, implemented via an Elite-Guided Opposition-Based Learning (EOL) mechanism. This operator establishes a dynamic center of symmetry guided by the current global best solution, $I_{\text {Best}}$, to generate new candidate solutions. The scope of this symmetric exploration is controlled by an adaptive scaling factor, $m_{\text {obl}}$, as defined in Eq. 9, while the new position is calculated according to Eq. 10.

9 $$\begin{aligned} m_{\text {obl}}= & (1 + \text {iter}/\text {MaxIter})^{c1} \end{aligned}$$

10 $$\begin{aligned} I'_{kj}= & C_j + (C_j - I_{\text {Best},j}) / m_{\text {obl}} - (I_{kj} - C_j) / m_{\text {obl}} \end{aligned}$$

This is complemented by an *asymmetric perturbation operator*, which acts as a powerful instrument for breaking convergence inertia. This operator is designed to introduce a high-magnitude, non-patterned stochastic shock using a t-distribution, which is particularly effective for escaping local optima. Its behavior is governed by a dynamically evolving degrees of freedom parameter, $t_r$, shown in Eq. 11. When probabilistically triggered, a new position is generated using Eq. 12.

11 $$\begin{aligned} t_r= & \exp (1 + (1 + \text {iter}/\text {MaxIter})^{c2}) \end{aligned}$$

12 $$\begin{aligned} I''_{kj}= & C_j + (I_{\text {Best},j} - C_j) \cdot \text {rand}() \cdot S_j \end{aligned}$$

where $S_j$ is a random sample drawn from a t-distribution with $t_r$ degrees of freedom. The dynamic interplay between these two operators, as detailed in the pseudocode in Algorithm 1 (lines 21-34), endows E-IVYA with a superior diversity maintenance capability.

### Dynamic perturbation escape mechanism based on elite differential mutation

To address the critical challenge of search stagnation, where individuals become trapped in local optima, we designed a Dynamic Perturbation Escape Mechanism based on Elite Differential Mutation. The core idea is to leverage the collective intelligence of the elite subpopulation to guide the search out of a suboptimal region, rather than resorting to blind or random perturbations.

The mechanism is triggered when a stagnation counter, $\text {stagnation\_count}$, exceeds a predefined threshold, $\text {MaxStagnation}$. As outlined in Algorithm 1 (lines 42-52), the process involves three key steps. First, a Dynamic Elite Archive (DEA) is constructed by selecting the top $p\%$ of individuals from the current population:

13 $$\begin{aligned} DEA = \{I_i | I_i \in \text {Top}_p(\text {Pop})\} \end{aligned}$$

Second, inspired by the principles of Differential Evolution, an Elite Differential Perturbation Vector ($V_{\text {edp}}$) is generated using three distinct elite individuals ($I_{r1}, I_{r2}, I_{r3}$) randomly selected from the DEA:

14 $$\begin{aligned} V_{\text {edp}} = I_{r1} + F \cdot (I_{r2} - I_{r3}) \end{aligned}$$

where *F* is a scaling factor. Finally, this vector is applied to a targeted stagnated individual $I_i$ to calculate its new escape position:

15 $$\begin{aligned} I_{i}^{\text {new}} = I_i + V_{\text {edp}} \end{aligned}$$

This process creates a guided, high-potential leap away from the local trap. The differential vector, $F \cdot (I_{r2} - I_{r3})$, provides the necessary *exploratory* step, while the base vector $I_{r1}$ anchors the perturbation in a high-performance region, thereby maintaining a strong *exploitative* tendency. This intelligent fusion of exploration and exploitation significantly enhances the global search performance and overall robustness of E-IVYA.

### Adaptive movement strategy integrating sine-cosine algorithm principles

**Fig. 2:**
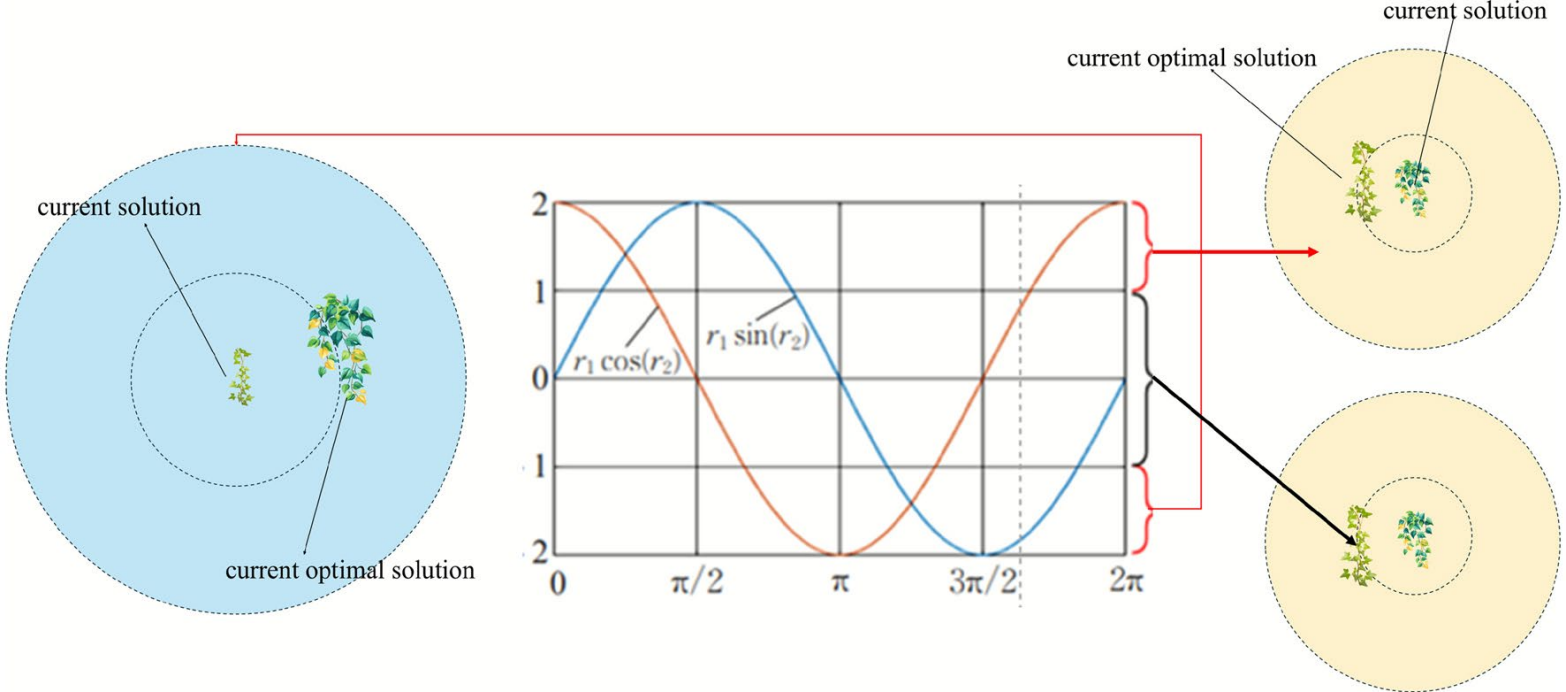
Adaptive Movement Strategy Integrating Sine-Cosine Algorithm Principles.

To address the relatively static exploration-exploitation balance in the original IVYA, we introduce a more adaptive individual movement strategy by integrating the core principles of the Sine-Cosine Algorithm (SCA). This enhancement, conceptually illustrated in Fig. 2, replaces the two original position update equations with new formulas that provide a dynamic and smooth transition between global and local search.

The selection between the local and global search modes still follows IVYA’s original $\beta$ condition logic. If the local growth condition is met, the new position $I_i^{\text {new}}$ is updated according to Eq. 16:

16 $$\begin{aligned} I_i^{\text {new}} = I_i + r_1 \cdot \sin (r_2) \odot (|N(1,D)| \odot (I_{ii} - I_i)) + r_1 \cdot \cos (r_3) \odot (N(1,D) \odot \Delta Gv_i) \end{aligned}$$

Otherwise, the individual executes a global exploratory movement regulated by the SCA mechanism, as shown in Eq. 17:

17 $$\begin{aligned} I_i^{\text {new}} = I_i + r_1 \cdot \sin (r_2) \odot (r_4 \cdot I_{\text {Best}} - I_i) + r_1 \cdot \cos (r_3) \odot \Delta Gv_i \end{aligned}$$

The key to this adaptive behavior lies in the SCA adjustment parameters. Parameter $r_1$ is a dynamic factor calculated as $r_1 = a - a \cdot (\text {iter}/\text {MaxIter})$ (where $a=2$), which linearly decreases over iterations. This systematically reduces the movement step size, guiding the algorithm from broad exploration to fine-grained exploitation. Parameters $r_2, r_3 \in [0, 2\pi ]$ and $r_4 \in [0, 1]$ are random numbers that introduce diverse oscillatory characteristics and stochasticity to the search process. This adaptive update is performed for each individual in the main loop of the algorithm, as shown in Algorithm 1 (line 16), achieving a significantly more flexible and efficient dynamic equilibrium between the search phases.

### E-IVYA: an enhanced Ivy algorithm

The Enhanced Ivy Algorithm (E-IVYA) significantly refines the original IVYA by integrating three core mechanisms that operate synergistically. This integration is designed to overcome the inherent limitations of IVYA when addressing complex optimization problems. These mechanisms work together to elevate the algorithm’s overall performance.

First, the Elite-Guided Opposition-Based Learning and Adaptive Perturbation Enhancement Strategy systematically bolsters population diversity. It achieves this by combining elite-information-guided opposition-based learning with probabilistic t-distribution perturbations. This strategy broadens the algorithm’s effective search range and mitigates the risk of premature convergence. Second, the Stagnation Response and Adaptive Growth Direction Adjustment Mechanism markedly improves the algorithm’s capacity to escape local optimal regions. This is accomplished by monitoring the algorithm’s convergence state in real-time and proactively adjusting the growth update direction of certain individuals when search stagnation is detected, thereby re-energizing the exploration process. Third, the Adaptive Movement Strategy Integrating Sine-Cosine Algorithm Principles optimizes IVYA’s individual movement methodology. It introduces dynamically varying adjustment parameters that change with iterations and incorporates the periodic oscillatory characteristics of trigonometric functions. This leads to a more flexible and efficient dynamic equilibrium between global exploration and local exploitation. The organic combination and synergistic effect of these three key mechanisms endow E-IVYA with stronger global optimization capabilities, superior convergence characteristics, and increased robustness when solving complex continuous optimization problems. This effectively addresses the potential deficiencies of the original IVYA.

**Algorithm 1:**
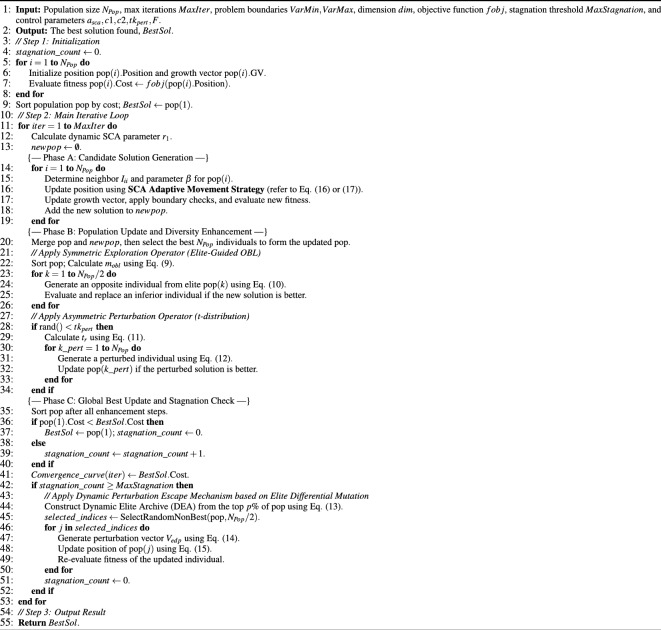
The pseudo-code of the E-IVYA

### Computational complexity analysis of E-IVYA

An analysis of the computational complexity is essential for evaluating the efficiency of the proposed E-IVYA. The complexity of the original IVYA is primarily determined by population initialization, fitness evaluation, and sorting, resulting in an overall complexity of $O(\text {MaxIter} \cdot (N_{\text {Pop}} \log N_{\text {Pop}} + N_{\text {Pop}} \cdot D + N_{\text {Pop}} \cdot f))$.

The E-IVYA introduces three enhancement mechanisms. The SCA-based movement strategy and the EOL mechanism involve vector operations, adding a complexity of $O(N_{\text {Pop}} \cdot D)$ per iteration. The elite differential mutation escape mechanism involves sorting to build the elite archive ($O(N_{\text {Pop}} \log N_{\text {Pop}})$) and performing vector operations on a subset of the population. Therefore, the overall computational complexity of E-IVYA for a complete run remains in the same order as the original, approximated as $O(\text {MaxIter} \cdot (N_{\text {Pop}} \log N_{\text {Pop}} + N_{\text {Pop}} \cdot D + N_{\text {Pop}} \cdot f))$.

While the enhancement strategies introduce a modest increase in computational overhead per iteration compared to the baseline IVYA, this cost is comparable to that of many other state-of-the-art metaheuristic algorithms. As demonstrated by the experimental results, this slight increase in computational cost yields a significant improvement in optimization performance, particularly in convergence speed and solution accuracy. This represents a favorable trade-off, especially for tackling complex and computationally expensive AI problems where solution quality is paramount.

## Experimental environment and setting

### Experimental fundamentals

To rigorously evaluate the comprehensive performance of the E-IVYA algorithm proposed in this study, all experiments were conducted on a unified computing platform to ensure a fair comparison. The experimental environment consisted of a Windows 11 operating system, with the algorithm implementation and simulations based on MATLAB R2021b. The hardware configuration of the computer used was an Intel(R) Core(TM) i7-11700K CPU @ 3.60GHz, equipped with 32 GB of RAM. This evaluation selected two internationally recognized standard test suites, IEEE CEC 2014 and IEEE CEC 2017, to examine the performance of E-IVYA on optimization problems with varying levels of complexity and different characteristics ^43^. Considering the inherent stochastic nature of metaheuristic algorithms, this study employs the non-parametric *Wilcoxon signed-rank test*. This statistical method is utilized to analyze the significance of performance differences among the algorithms, thereby providing stronger statistical support for the experimental conclusions.

### Comparison algorithms

To comprehensively and objectively validate the performance and effectiveness of the proposed E-IVYA algorithm, this study selected a series of representative metaheuristic algorithms for a comprehensive performance comparison. The selection of these algorithms covers the original baseline algorithm, classic metaheuristics, advanced variants of differential evolution, and several recently proposed novel algorithms. This approach aims to rigorously evaluate the competitiveness of E-IVYA from multiple perspectives. The specific comparison algorithms selected are as follows:

*Original Baseline Algorithm* Original Ivy Algorithm (IVYA) is included as the most important baseline for comparison ^28^. The purpose is to directly and clearly measure the comprehensive performance improvement brought by the various enhancement strategies proposed in this study.

*Classic and Widely-Used Metaheuristic Algorithms* Particle Swarm Optimization (PSO) ^18^, Grey Wolf Optimizer (GWO) ^44^, Differential Evolution (DE) ^17,45^, and Reptile Search Algorithm (RSA) were chosen because they are classic methods in the optimization field that have been long-validated, are widely used, and have recognized performance ^46^. Comparing E-IVYA with these algorithms allows for an evaluation of its performance advantages against standard metaheuristics.

*Advanced Differential Evolution Variants:* Adaptive Differential Evolution with Optional External Archive (JADE) and Success-History based Adaptive DE with Linear Population Size Reduction (LSHADE) are included ^47,48^. Differential Evolution is a powerful paradigm for solving continuous optimization problems. As highly successful variants with advanced adaptive mechanisms, JADE and LSHADE represent a high level of performance in the field of evolutionary algorithms. Comparison with them helps to test the competitiveness of E-IVYA against top-tier evolutionary algorithms when tackling complex optimization problems.

*Recently Proposed Novel Optimization Algorithms:* Animated Oat Optimization (AOO) algorithm ^49^, Liver Cancer algorithm (LCA) ^50^, and Water Uptake and Transport in Plants (WUTP) algorithm are selected ^51^. To examine the performance of E-IVYA in comparison to recent trends in optimization algorithm research, this study selected these newer algorithms. Comparing with them allows for a better assessment of E-IVYA’s novelty and its advancement within the current context of algorithm development.

By conducting a comprehensive comparison with the carefully selected algorithms listed above, the performance level of E-IVYA can be more reliably positioned. This also enables a deeper analysis of the specific contributions of its various enhancement mechanisms under different types of optimization challenges. The algorithm parameter Settings are shown in Table 1.

**Table 1:** Parameter settings for the proposed E-IVYA and the comparison algorithms.

Algorithm	Parameter	Value
E-IVYA	SCA initial control parameter (*a*)	2
	DE scaling factor (*F*)	0.5
	Elite archive percentage (*p*)	0.1
	Stagnation threshold (MaxStagnation)	30
	Asymmetric perturbation probability ($t_k$)	0.05
	Symmetric exploration exponent (*c*1)	2
	Asymmetric exploration exponent (*c*2)	2
IVYA	Behavioral switch parameter ($\beta$)	$1 + \text {rand}/2 \in [1, 1.5]$
PSO	Acceleration constants	0.2
GWO	$\vec {a}$	linearly decreased from 2 to 0
DE	crossover rate	0.8
	scale factor primary	0.6
	use mutation scheme	1
	use sorted selection	0
RSA	Evolutionary Sense	$2 \times \text {randn} \times (1-(iter/maxiter))$
	Sensitive parameter controlling the exploration accuracy	0.05
	Sensitive parameter controlling the exploitation accuracy	0.1
JADE	c	0.1
	UCR	0.5
	UF	0.5
	top	1
LSHADE	$r^{N^{init}}$	30
	$r^{arc}$	1.4
	p	0.11
	Historical memory size (H)	5
AOO	Levy flight distribution parameter ($\beta$)	1.5
	Decay factor exponent	3
	Elite group divisor	10
LCA	Tumor growth rate (*p*)	2/3
	Levy flight distribution parameter ($\beta$)	1.5
WUTP	Alternate parameter (*p*)	0.5
	Probability threshold (*rr*)	0.1
	Control parameter ($\chi$)	0.5

### Evaluation metrics

In this study, four main metrics were employed to evaluate the performance of the proposed E-IVYA algorithm: diversity, exploration, exploitation, and the convergence curve ^52–54^. These metrics are designed to assess the algorithm’s ability to explore the search space, its performance in global search (exploration), and its effectiveness in local search within identified potential optimal regions (exploitation).

*Diversity Evaluation.* Evaluating the population diversity of E-IVYA is crucial for measuring its ability to effectively explore and exploit the search space. This diversity is quantified using Equations (X) and (Y):

18 $$\begin{aligned} \text {Diversity}= & \frac{1}{N_{Pop}} \sqrt{ \sum _{i=1}^{N_{Pop}} \sum _{j=1}^{D} (I_{ij} - \bar{I}_j)^2 } \end{aligned}$$

19 $$\begin{aligned} \bar{I}_j= & \frac{1}{N_{Pop}} \sum _{i=1}^{N_{Pop}} I_{ij} \end{aligned}$$

Here, $N_{Pop}$ is the total population size, *D* is the problem dimension, and $\bar{I}_j$ is the average position of the population in the *j*-th dimension.

*Exploration and Exploitation Evaluation* Based on population diversity, the exploration and exploitation behaviors can be defined by Equations (Z) and (W):

20 $$\begin{aligned} \text {Exploration}= & \frac{\text {Diversity}}{\text {Diversity}_{max}} \end{aligned}$$

21 $$\begin{aligned} \text {Exploitation}= & \frac{|\text {Diversity}_{max} - \text {Diversity}|}{\text {Diversity}_{max}} \end{aligned}$$

where $\text {Diversity}$ is the population diversity of the current iteration, and $\text {Diversity}_{max}$ is the maximum diversity value recorded throughout the entire run.

*Convergence Curve* The convergence curve is key to evaluating an algorithm’s performance, as it visually demonstrates the rate at which the algorithm converges towards the optimal solution during the iterative process. Typically, the convergence curve plots the relationship between the objective fitness value and the number of iterations. The slope of the curve can serve as an indicator of the convergence speed towards the optimum. In the initial phase of the curve, a significant drop usually indicates exploration activities, whereas subsequent phases show more gradual improvements, representing the exploitation capability of the search process.

## Results and discussion

This chapter aims to comprehensively evaluate the performance of the proposed Enhanced Ivy Algorithm (E-IVYA), based on the experimental environment and setup detailed in Section 5. To clearly demonstrate the advantages of E-IVYA, this chapter is organized as follows: First, Experiment 1 provides a qualitative analysis of E-IVYA’s search behavior, with a focus on its population diversity and exploration-exploitation dynamics. Subsequently, Experiment 2 and Experiment 3 present extensive quantitative comparisons of E-IVYA’s performance against other algorithms on the IEEE CEC 2014 and CEC 2017 benchmark test suites. Building on this, Experiment 4 validates the significance of the performance differences through rigorous statistical tests.

### Experiment 1: diversity, exploration and exploitation analysis

*Diversity Analysis* Population diversity is a critical metric for evaluating a metaheuristic algorithm’s capacity to explore the search space thoroughly and avoid premature convergence to local optima. This analysis verifies the effectiveness of E-IVYA’s enhancement mechanisms in maintaining exploratory vitality. To assess this capability, three representative functions were selected: the unimodal function F1, the multimodal function F8, and the composite function F21. The experimental results indicate that E-IVYA demonstrates a robust ability to maintain population diversity throughout the iterative process. Particularly when dealing with complex multimodal and composite functions, although diversity naturally decreases as the algorithm converges on promising regions, E-IVYA periodically injects new vitality into the population. This capability is primarily attributed to the ’Elite-Guided Opposition-Based Learning and Adaptive Perturbation Enhancement Strategy,’ which allows the population diversity to recover from temporary declines, thereby preventing search stagnation. The diversity change curves for these functions are presented in Fig. 7.

*Exploration and Exploitation Analysis* The balance between exploration and exploitation is a core indicator of a metaheuristic algorithm’s performance. This section evaluates E-IVYA’s ability to dynamically coordinate these two search behaviors throughout the optimization process, using the unimodal function F1, multimodal function F8, and composite function F23 for assessment. Compared to the original IVYA, E-IVYA exhibits a significantly improved ability to manage this balance. When handling unimodal problems, it transitions rapidly from exploration to exploitation for fast convergence. Conversely, when facing more complex multimodal and composite functions, it sustains a more effective equilibrium between the two behaviors. This is attributed to the ’Adaptive Movement Strategy Integrating SCA Principles,’ which facilitates a high level of exploration in the early stages and smoothly shifts to exploitation for fine-tuning solutions later. While a perfect balance remains a challenge in high-dimensional problems, the dynamic adjustment capability of E-IVYA is demonstrably to that of the baseline algorithm, as shown in Fig. 8.

In summary, the experimental results clearly demonstrate that the enhancement strategies integrated into E-IVYA synergistically improve its performance. The ’Elite-Guided Opposition-Based Learning and Adaptive Perturbation Enhancement Strategy’ is principally responsible for maintaining high population diversity. Concurrently, the ’Adaptive Movement Strategy Integrating SCA Principles,’ through its dynamic parameter $r_1$, achieves an effective and smooth transition between exploration and exploitation. Furthermore, the ’Stagnation Response and Adaptive Growth Direction Adjustment Mechanism’ provides a critical escape capability, ensuring the continuity of the search process when the algorithm stagnates. The collective effect of these mechanisms significantly enhances E-IVYA’s robustness and efficiency in solving complex optimization problems.

### Experiment 2: algorithm performance comparison on IEEE CEC 2014

To evaluate the performance of E-IVYA under different dimensions, this section presents a comprehensive comparison with a series of comparison algorithms on the IEEE CEC 2014 test suite. The experiments were conducted under both 50-dimension (50D) and 100-dimension (100D) settings. All algorithms were run independently for 30 rounds in a unified environment, with 500 iterations per round. The detailed quantitative results of the experiments are recorded in Table 4 (50D) and Table 5 (100D), while the convergence curves for some representative functions are shown in Fig. 9 (50D) and Fig. 10 (100D).

The experimental results clearly show that E-IVYA demonstrates a leading performance on most test functions, leading in the overall rankings under both 50D and 100D challenges. Its superior performance can be attributed to the synergistic effect of its three innovative mechanisms. For unimodal functions, the ’Adaptive Movement Strategy Integrating SCA Principles’ ensures fast and precise convergence through refined local search. For the more complex multimodal, hybrid, and composite functions, the ’Elite-Guided Opposition-Based Learning and Adaptive Perturbation Enhancement Strategy’ and the ’Stagnation Response Mechanism’ work together to maintain population diversity and effectively escape local optima. The organic combination of these mechanisms enables E-IVYA to efficiently balance exploration and exploitation, thereby consistently obtaining high-quality solutions in various complex optimization problems.

### Experiment 3: algorithm performance comparison on IEEE CEC 2017

To further examine the performance and robustness of E-IVYA on more challenging optimization problems, this subsection utilizes the IEEE CEC 2017 benchmark test suite for evaluation. This test suite also includes unimodal, multimodal, hybrid, and composite functions, but it introduces more complex rotations, shifts, and combinations in their characteristics, posing higher demands on the comprehensive optimization capabilities of an algorithm. The comparative analysis in this section continues with the previous experimental setup, also conducted under 50-dimension (50D) and 100-dimension (100D) settings.

The detailed quantitative comparison results are presented in Table 6 (50D) and Table 7 (100D), with the corresponding convergence curves shown in Fig. 11 (50D) and Fig. 12 (100D). The experimental results demonstrate that E-IVYA continues to exhibit strong optimization capabilities in the more complex CEC 2017 test environment, achieving the best fitness values on most functions in both dimensions. This success is attributed to the robust balance between exploration and exploitation in its design. It is noteworthy that the group of comparison algorithms includes advanced differential evolution variants such as JADE and LSHADE. E-IVYA still shows a significant advantage in competition with these top-tier algorithms, with its performance being particularly outstanding in high-dimensional scenarios.

The excellent performance of E-IVYA on the CEC 2017 test suite further confirms the effectiveness of its combined enhancement strategies. The ’Elite-Guided Opposition-Based Learning and Adaptive Perturbation Enhancement Strategy’ maintains necessary population diversity by continuously optimizing the population structure, while the ’Adaptive Movement Strategy Integrating SCA Principles’ and the ’Stagnation Response Mechanism’ together ensure the algorithm’s efficient exploration and stable convergence in complex search landscapes. The results in Tables 8 and 9 consistently demonstrate that E-IVYA achieves remarkably low p-values when compared against the vast majority of algorithms on both the CEC 2014 and the more complex CEC 2017 test suites. At the conventional significance levels of $\alpha =0.1$ and $\alpha =0.05$, nearly all* p*-values are well below the threshold, allowing for the rejection of the null hypothesis. This indicates that the performance advantage of E-IVYA is statistically significant and not a result of random chance. These findings provide strong statistical support for the conclusion that the proposed E-IVYA algorithm is significantly superior to the other comparison algorithms selected for this study. These results provide strong evidence that E-IVYA, as a robust optimization method, has strong potential for solving challenging and complex optimization problems.

### Experiment 4: statistical experiments

To statistically validate the performance advantage of E-IVYA over its counterparts, this section employs the Wilcoxon signed-rank test ^55^. This non-parametric method is well-suited for evaluating the differences between paired samples, as it does not assume a normal data distribution. The analysis utilizes the data from the 30 independent runs for each algorithm on the IEEE CEC 2014 and IEEE CEC 2017 test suites, ensuring consistency with the previous experimental sections.

Tables 8 and 9 present the results of the pairwise comparisons between E-IVYA and each of the other algorithms, detailing the calculated *p*-values. The fundamental argument of this study is that E-IVYA’s performance is significantly superior. In this context, a *p*-value below a preset significance level ($\alpha$) permits the rejection of the null hypothesis–which posits no significant performance difference–and thus confirms that E-IVYA’s advantage is statistically significant.

The results in Tables 8 and 9 consistently demonstrate that E-IVYA achieves remarkably low *p*-values when compared against the vast majority of algorithms on both the CEC 2014 and the more complex CEC 2017 test suites. Consequently, at the conventional significance levels of $\alpha =0.1$ and $\alpha =0.05$, the superiority of E-IVYA receives strong statistical support. These analytical findings support the conclusion that the proposed E-IVYA algorithm is significantly superior, in terms of performance, to the other comparison algorithms selected for this study.

### Experiment 5: ablation study of E-IVYA components

To rigorously validate the individual contribution of each enhancement mechanism proposed in this paper, an ablation study was conducted. This experiment aims to deconstruct the E-IVYA framework and quantify the performance impact of its three core components: the Dynamic Perturbation Framework (DPF), the Elite Differential Mutation (EDM) escape mechanism, and the Adaptive Movement Strategy integrating SCA principles (SCA). For this purpose, we compared the performance of the baseline IVYA, the final integrated E-IVYA, and three intermediate variants, each incorporating only a single enhancement mechanism. The experiments were performed on ten representative CEC 2014 benchmark functions and the challenging Credit-Card Fraud dataset for GAN hyperparameter optimization.

The detailed quantitative results of this ablation study are presented in Table 10 and Figs. 13 and 14. The findings clearly indicate that each proposed mechanism provides a distinct and positive contribution to the algorithm’s performance. As shown in the table, all three single-mechanism variants (IVYA+DPF, IVYA+EDM, and IVYA+SCA) consistently outperform the original IVYA across all test platforms. For instance, on the complex composite function F21, the baseline IVYA achieved a mean fitness of $6.66 \times 10^6$, whereas the IVYA+EDM and IVYA+SCA variants improved this result to $1.37 \times 10^6$ and $1.27 \times 10^5$, respectively. This highlights the significant role of the elite-guided escape strategy and the adaptive movement mechanism in navigating complex, multi-modal landscapes. This trend is further confirmed in the GAN optimization task, where all variants surpassed the baseline F1-Score of 0.82, with IVYA+EDM achieving a notable score of 0.85.

Most importantly, the results demonstrate a clear and significant synergistic effect among the three strategies. The complete E-IVYA model, which integrates all components, achieved the best performance on every single test case, significantly surpassing all intermediate variants. On the unimodal function F1, E-IVYA’s result ($3.35 \times 10^6$) is an order of magnitude better than the best-performing variant, IVYA+EDM ($1.70 \times 10^7$), showcasing a substantial improvement in convergence precision. This synergistic behavior can be attributed to the complementary roles of the mechanisms: the DPF maintains a healthy population diversity, providing a robust foundation for the SCA’s adaptive exploration and exploitation, while the EDM acts as a crucial failsafe, providing a guided escape from local optima that may still temporarily trap the search. In conclusion, this ablation analysis provides compelling evidence that the performance superiority of E-IVYA is not due to a single dominant component, but rather the well-designed, synergistic integration of all three enhancement strategies, validating the core design philosophy of this work.

### Experiment 6: computational cost analysis

To supplement the theoretical complexity analysis (Section 4.4), an empirical runtime analysis was conducted. This experiment aims to rigorously quantify the practical “wall-clock time” overhead introduced by the enhancement mechanisms. For this purpose, we compared the average runtime of the baseline IVYA, the final integrated E-IVYA, and the key competitors (PSO and JADE). The experiments were performed on three representative CEC 2014 benchmark functions (F1, F8, and F21 at 50D) and the computationally intensive Credit-Card Fraud dataset for GAN hyperparameter optimization. All tests were executed on the unified hardware platform detailed in Section 5.1.

The detailed quantitative results of this runtime analysis are presented in Table 11. The findings clearly indicate that E-IVYA introduces a modest and predictable increase in computational cost, which is consistent with its enhanced search capabilities. As shown in the table, on the CEC 2014 benchmarks, E-IVYA required approximately *1.36x to 1.37x* the runtime of the original IVYA, depending on the function’s complexity. For instance, on the composite function F21, the baseline IVYA required 81.33 seconds, whereas the complete E-IVYA model required 110.61 seconds. This additional overhead is an expected consequence of its three synergistic mechanisms (DPF, EDM, and SCA), which perform additional calculations per iteration (e.g., elite archive construction, t-distribution sampling, and trigonometric updates). Notably, this computational cost is comparable to other advanced algorithms like JADE, which also required more time (e.g., 1.14x) than the baseline IVYA.

Most importantly, the results demonstrate a clear and acceptable *trade-off between solution quality and computational cost*, which we explicitly acknowledge in the Limitations section. The baseline IVYA and classic PSO are faster (e.g., 1.00x and 0.81x-0.84x on CEC tasks, respectively), but this speed is achieved at the significant expense of performance, leading to premature convergence. The moderate runtime increase of E-IVYA is the necessary investment for its superior convergence, its robust ability to escape local optima (validated in *Experiment 5*), and its significantly better final solutions. This trend is further confirmed in the GAN optimization task, where a *1.26x* increase in runtime delivered the best-in-class F1-Score. In conclusion, this runtime analysis provides compelling evidence that the performance superiority of E-IVYA is not achieved at a prohibitive computational cost; rather, it represents a well-justified and highly favorable efficiency trade-off for tackling complex, real-world optimization problems.

### Experiment 7: parameter sensitivity analysis

To address the reviewer’s request regarding parameter stability and to further validate the robustness of E-IVYA, we conducted a sensitivity analysis on the two key control parameters introduced by the Elite Differential Mutation (EDM) mechanism: the elite archive percentage *p* and the scaling factor *F*. We employed a One-At-a-Time (OAT) methodology, varying one parameter while keeping the other at its default value. The analysis was performed on the three representative CEC 2014 functions (F1, F8, and F21) used in the previous analyses.

The results for the elite archive percentage *p* (while holding $F=0.5$) are presented in Fig. 15a, b, and c. The findings are highly conclusive. On the unimodal F1 and composite F21 functions (Fig. 15a, c), the default value of $p=0.1$ (red line) significantly outperforms all other settings. A slightly smaller value of $p=0.05$ (magenta line) provides the second-best performance. Critically, any value of $p \ge 0.2$ (blue, cyan, green lines) causes a dramatic degradation in performance, leading to premature stagnation. This strongly suggests that a large *p* “pollutes” the Dynamic Elite Archive (DEA) with non-elite solutions, which in turn degrades the quality of the differential perturbation vector $V_{edp}$. While the algorithm is less sensitive on the multimodal F8 (Fig.  15b), $p=0.1$ and $p=0.05$ still hold a marginal advantage. This analysis confirms that $p=0.1$ is an optimal and robust “sweet spot,” providing sufficient diversity for the mutation vector without compromising its elite-guiding capability.

The convergence curves for varying scaling factor *F* (while holding $p=0.1$) are presented in Fig. 16a, b, and c. This parameter controls the magnitude of the differential perturbation and presents a clear trade-off. On the complex, high-dimensional functions (F1 and F21, Fig.  16a, c), a very small factor of $F=0.3$ (blue line) provides insufficient perturbation and leads to the worst performance. Larger values, such as $F=0.8$ (green line) or $F=1.0$ (magenta line), demonstrate strong exploratory power, with $F=0.8$ achieving the best final result on F1 and F21. However, on the multimodal F8 (Fig.  16b), our default value of $F=0.5$ (red line) achieves the best (albeit marginal) result, indicating superior performance in navigating complex multimodal landscapes without excessive disruption. This confirms that our default choice of $F=0.5$, a standard and effective value in Differential Evolution literature, provides the most *robust and well-balanced performance* across all three distinct function types, validating it as a strong default setting.

In summary, this sensitivity analysis confirms that E-IVYA is not overly sensitive to its key parameters, and the default values chosen in Table 1 are well-justified, providing robust and effective performance across a diverse range of problem landscapes.

## Optimizing GAN parameters to improve imbalanced data classification

### E-IVYA-GAN: a framework for gan hyperparameter optimization

**Fig. 3:**
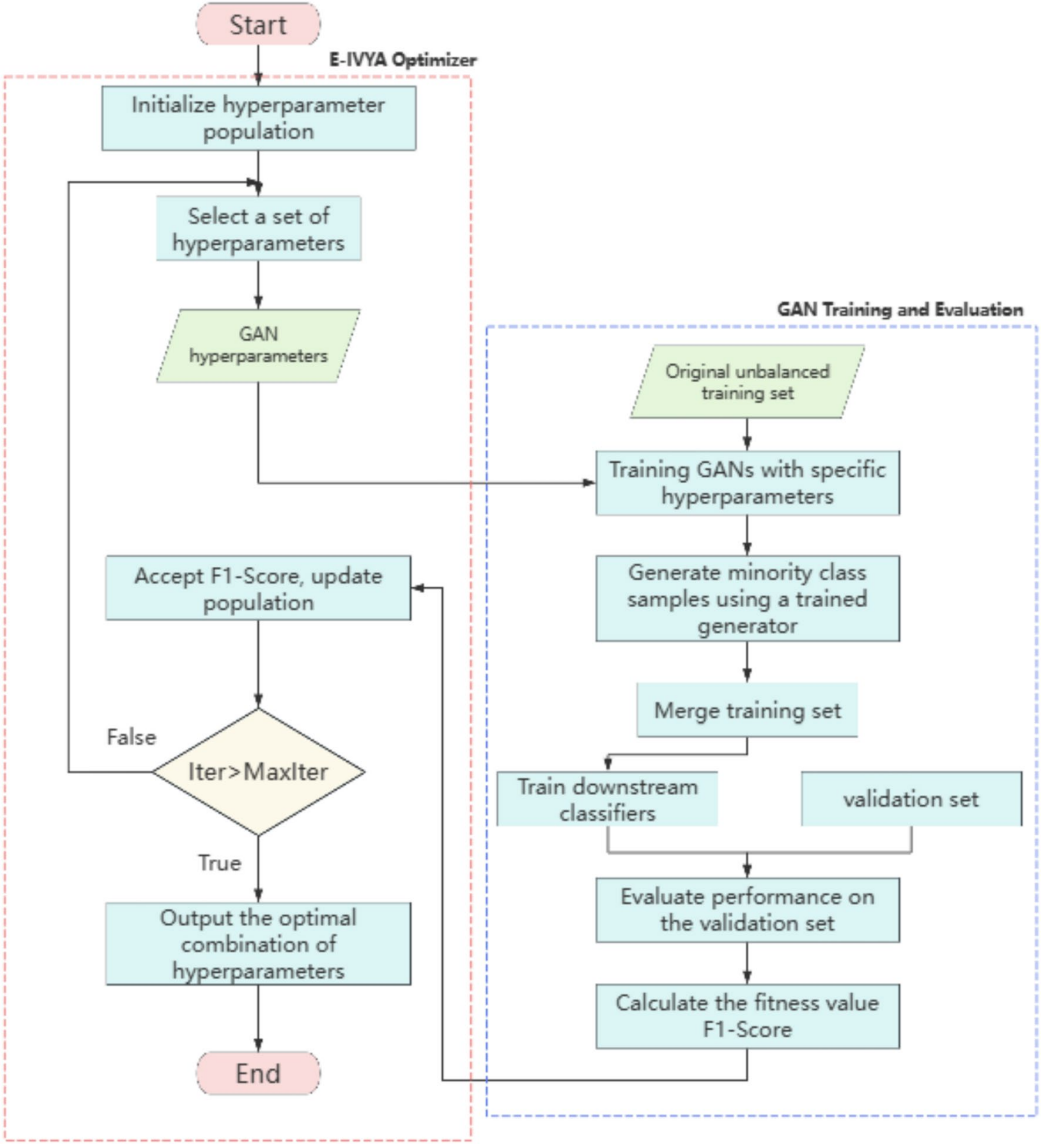
Flowchart of the E-IVYA-GAN hyperparameter optimization framework.

To leverage the powerful optimization capabilities of E-IVYA for solving the complex hyperparameter selection problem in GAN training, this study proposes an integrated optimization framework named E-IVYA-GAN. This framework aims to automatically and efficiently search for the optimal combination of GAN hyperparameters. Its ultimate goal is to enhance the quality and diversity of minority-class data samples generated by the GAN, thereby significantly improving the performance of a downstream classifier on imbalanced data classification tasks. Essentially, E-IVYA-GAN formalizes the process of GAN hyperparameter tuning as a complex optimization problem that can be solved by a metaheuristic algorithm. The overall workflow of this framework is illustrated in Fig. 3. The table of hyperparameters to be optimized for GAN is shown in Table 2.

#### Optimization framework workflow and individual encoding

Within this framework, the GAN hyperparameter optimization problem must first be mapped into a format that E-IVYA can solve. Specifically, a set of GAN hyperparameters to be optimized is encoded as an “ivy individual” within the E-IVYA population. Each individual is a *D*-dimensional real-valued vector $I = (h_1, h_2, \dots , h_D)$, where each dimension $h_j$ corresponds to a specific hyperparameter (e.g., generator learning rate $lr_G$, discriminator learning rate $lr_D$, Adam optimizer’s $\beta _1$ parameter, etc.). E-IVYA will search for the optimum within the predefined search space for these hyperparameters, which is defined by *VarMin* and *VarMax*.

The overall optimization process of the E-IVYA-GAN framework follows a standard metaheuristic optimization loop:

1. E-IVYA initializes a population of $N_{Pop}$ individuals, each representing a random combination of hyperparameters.
2. Each individual in the population is evaluated using a carefully designed fitness function to quantify its quality.
3. Based on the fitness values of all individuals, E-IVYA applies its unique enhanced growth and update strategies (as described in Sections 3.1-3.3) to generate a new generation of hyperparameter combinations.
4. Steps (2) and (3) are repeated until a preset maximum number of iterations is reached or other termination criteria are met.

Finally, the algorithm outputs the individual with the best fitness value, which represents the optimal GAN hyperparameter combination.

**Table 2:** Hyperparameters Optimized by E-IVYA for GANs, their Search Ranges, and Rationale.

Hyperparameter	Symbol	Search Range [Min, Max]	Rationale
Generator learning rate	$lr_G$	$[1 \times 10^{-5}, 1 \times 10^{-3}]$	Covers values commonly found in GAN literature for stable training.
Discriminator learning rate	$lr_D$	$[1 \times 10^{-5}, 1 \times 10^{-3}]$	Symmetric to $lr_G$; allows for exploring both balanced and unbalanced rates.
Adam optimizer beta1	$\beta _1$	[0.1, 0.9]	Encompasses the default (0.9) and GAN-specific stable values (e.g., 0.5).
Latent vector dimension	$z_{\text {dim}}$	[50, 200] (Integer)	Standard range for balancing model capacity and training efficiency.

#### Fitness function design

The design of the fitness function is the absolute core of the E-IVYA-GAN framework. It builds a bridge between the optimizer (E-IVYA) and the artificial intelligence task (improving imbalanced data classification performance). The key objective of this fitness function is to accurately quantify the ultimate effectiveness of a given set of GAN hyperparameters in solving the class imbalance problem. Therefore, it does not evaluate the internal loss function values from the GAN’s training process (such as generator or discriminator loss). Instead, it assesses the actual contribution to the performance of an independent, downstream classification task after using the GAN to generate data.

For any individual in the E-IVYA population (i.e., a set of GAN hyperparameters to be evaluated), its fitness value is calculated through the following rigorous, automated evaluation process:

1. *GAN Model Training* Using the hyperparameter combination represented by the current individual, a GAN model is trained on a fixed architecture using the original imbalanced training dataset.
2. *Synthetic Sample Generation* After the GAN is trained, its generator is used to create a specified number of new, synthetic data samples for the minority class.
3. *Dataset Reconstruction* The generated synthetic minority samples are merged with the original training dataset to construct a new, more class-balanced augmented training set.
4. *Downstream Classifier Training* A downstream classifier with a fixed architecture and parameters (e.g., a standard convolutional neural network) is trained on this augmented training set. Keeping the classifier constant ensures that all comparisons only reflect the quality of the GAN hyperparameters.
5. *Performance Validation and Fitness Calculation* The trained downstream classifier is tested on an independent validation set, which was not used for either GAN or classifier training. A metric suitable for imbalanced data evaluation, such as the F1-Score, G-Mean, or Balanced Accuracy of the minority class, is used as the final fitness value for the individual. A higher classification performance metric represents a better fitness, indicating that the corresponding GAN hyperparameter combination is more effective.

### Experimental setup

This section aims to validate the effectiveness of the E-IVYA-GAN framework in optimizing GANs for imbalanced data classification tasks through a series of carefully designed experiments.

#### Datasets, model architectures, and implementation details

*Datasets*

To comprehensively evaluate the framework’s performance and generality, this study selected three public datasets with different characteristics, covering both tabular and image data types, as well as varying degrees of imbalance:

- *Credit-Card Fraud* This is a classic, highly imbalanced tabular dataset commonly used in fraud detection research. The minority class (fraudulent transactions) accounts for only 0.172% of the samples, posing a severe challenge for optimization algorithms and generative models.
- *Ecoli* This is a moderately imbalanced tabular dataset from the UCI repository, involving the classification of protein localization sites. Its minority class samples constitute approximately 5.97%, representing a common imbalance scenario in many bioinformatics applications.
- *Imbalanced MNIST* To test the framework’s performance on image data, we manually constructed an imbalanced version of the MNIST handwritten digit dataset. We designated the digit ’9’ as the minority class and merged the remaining digits (’0’-’8’) into the majority class, creating a controllable imbalanced image classification task.

Data Partitioning Strategy To ensure a rigorous and unbiased evaluation, a fixed data splitting strategy was applied to all three datasets *before* any experimentation. Each dataset was partitioned once into three independent, non-overlapping sets:

- A *Training Set (60% of data)*, which was used exclusively to train the GAN models.
- A *Validation Set (20% of data)*, which was used to evaluate the downstream classifier and provide the F1-Score as the fitness value to the E-IVYA optimizer.
- A *Held-Out Test Set (20% of data)*, which was completely isolated during the optimization process and used only once to report the final, unbiased performance metrics presented in Table 3.

All three sets (Training, Validation, and Test) maintained their original, imbalanced class distribution. This strict partitioning prevents any data leakage and ensures that the final reported results reflect the true generalization performance of the optimized framework.

*Model Architectures*

To ensure a fair comparison where all performance differences stem from different GAN hyperparameters rather than model structures, the network architectures of all models in this experiment were kept fixed:

- *GAN Models* For the two tabular datasets, Credit-Card Fraud and Ecoli, we used a standard GAN composed of Multi-Layer Perceptrons (MLPs). For the Imbalanced MNIST image dataset, we employed a classic Deep Convolutional Generative Adversarial Network (DCGAN).
- *Downstream Classifiers* For tabular data, a simple MLP classifier was used. For the MNIST image data, a Convolutional Neural Network (CNN) with a LeNet-5 architecture was used as the classifier.

*Hyperparameters to be Optimized*

The objective for E-IVYA and other comparison optimizers is to find the optimal combination for the following four key GAN hyperparameters, with their search ranges set as follows:

- Generator Learning Rate ($lr_G$): [1e-5, 1e-3]
- Discriminator Learning Rate ($lr_D$): [1e-5, 1e-3]
- Adam Optimizer’s beta1 parameter ($\beta _1$): [0.1, 0.9]
- Latent Vector Dimension ($z\_dim$):^50^

#### Implementation details for reproducibility

To ensure full reproducibility, several key implementation details were fixed for the GAN training and evaluation pipeline. First, all experiments were initialized using a global random seed of 42 to guarantee consistent results. Second, during each fitness evaluation performed by the optimizer (i.e., each call to the fitness function), the corresponding GAN model was trained on the original imbalanced training set using a batch size of 128 for a fixed 50 epochs. Finally, after this training, the generator was used to synthesize new minority-class samples. The number of generated samples was set to *equal the number of samples in the majority class*, thus creating a fully 1:1 balanced dataset for training the downstream classifier.

#### Baselines and comparison methods

To comprehensively measure the performance of the E-IVYA-GAN framework, we established several baselines and a series of challenging comparison methods. The baselines include training the classifier with *No Augmentation* (on the original imbalanced data) and using the classic *SMOTE* oversampling technique. For evaluating optimizer performance, E-IVYA is benchmarked against a diverse group on the GAN hyperparameter optimization task. This group includes *Random Search* as a non-intelligent benchmark; classic metaheuristics such as *PSO*, *GWO*, and the original *IVYA*; high-performance evolutionary algorithms, specifically the advanced DE variants *JADE* and *LSHADE*; and a specialized method for hyperparameter tuning, *Bayesian Optimization (BO)*.

#### Evaluation metrics

Considering the nature of class imbalance, the traditional accuracy metric can be misleading. Therefore, this experiment employs the following metrics, which are more suitable for imbalanced data classification, to evaluate the final performance of the downstream classifier:

- *F1-Score (Minority Class)* The harmonic mean of precision and recall for the minority class, serving as a core metric for evaluating imbalanced classification performance.
- *G-Mean* The geometric mean of the recall of the majority and minority classes, reflecting the model’s performance on both classes simultaneously.
- *Balanced Accuracy* The arithmetic mean of the recall of each class, also suitable for imbalanced scenarios.
- *AUC-PR* The area under the Precision-Recall (PR) curve. This metric is more reliable and informative than the traditional AUC-ROC when dealing with highly imbalanced data.

### Experimental results and analysis

This section aims to present and analyze the experimental results of the E-IVYA-GAN framework on imbalanced data classification tasks. We will demonstrate the effectiveness and superiority of E-IVYA in optimizing GAN hyperparameters through quantitative performance comparisons, analysis of the optimization process, and an in-depth evaluation of classifier performance.

#### Quantitative performance comparison and analysis

As presented in Table 3, the approach using E-IVYA to optimize GAN hyperparameters consistently achieves superior results across all three imbalanced datasets on key performance metrics, including F1-Score, G-Mean, and AUC-PR. The performance improvement is particularly pronounced on the highly imbalanced Credit-Card Fraud dataset, where E-IVYA obtains an F1-Score of 0.87, far surpassing the outcomes from both no data augmentation (0.56) and the traditional SMOTE method (0.71).

**Fig. 4:**
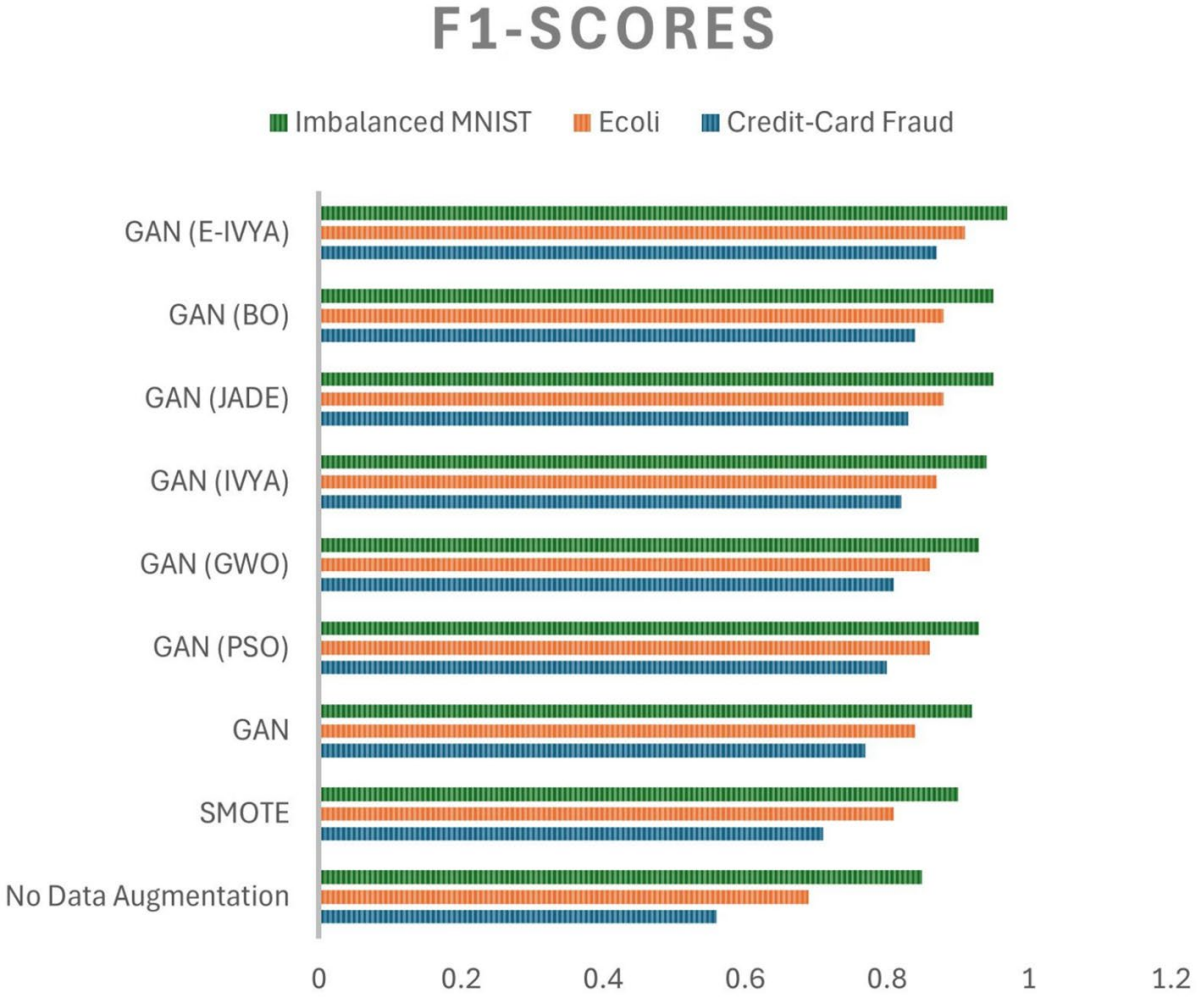
Comparison of the final classification performance (F1-Score) of different methods on various datasets.

E-IVYA also demonstrates a distinct advantage over other metaheuristic optimizers. It outperforms strong competitors such as JADE, LSHADE, and even the specialized Bayesian Optimization (BO) in final classification performance. Furthermore, the smaller standard deviation of its results indicates that E-IVYA is not only superior in performance but also highly stable in identifying high-quality hyperparameter combinations. These findings are visually corroborated by the bar chart in Fig. 4, where the bar representing ’GAN (E-IVYA)’ is consistently the highest for the F1-Score metric across all datasets, signifying its comprehensive lead.

These quantitative results provide strong evidence that E-IVYA can more effectively identify a set of hyperparameters that enables a GAN to generate high-quality, diverse minority-class samples compared to the other methods. This, in turn, significantly enhances the classification ability of the downstream classifier on imbalanced data.

#### In-depth analysis of optimization process and performance

**Fig. 5:**
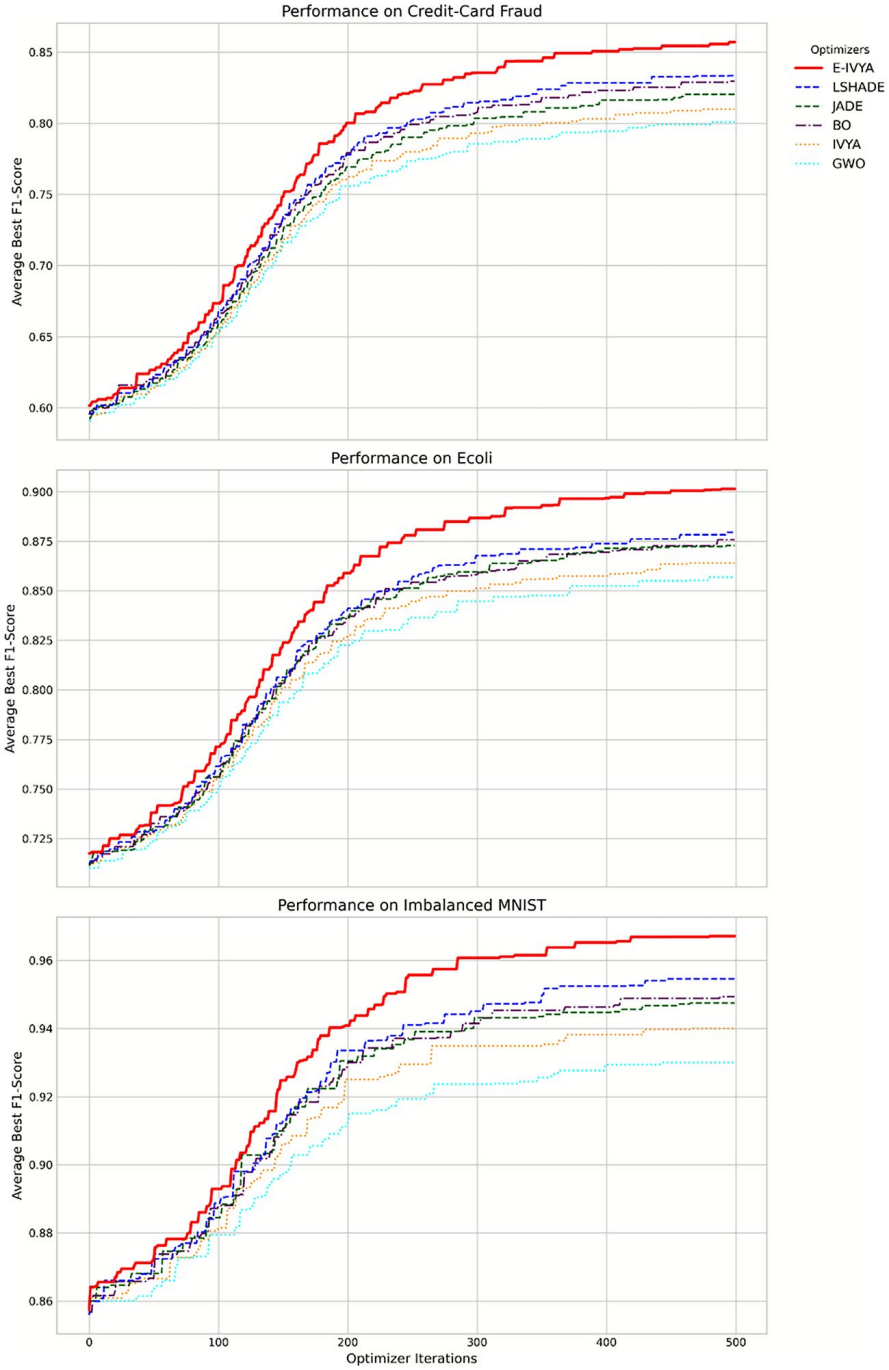
GAN training dynamics and performance convergence curves under E-IVYA optimization on various datasets.

**Fig. 6:**
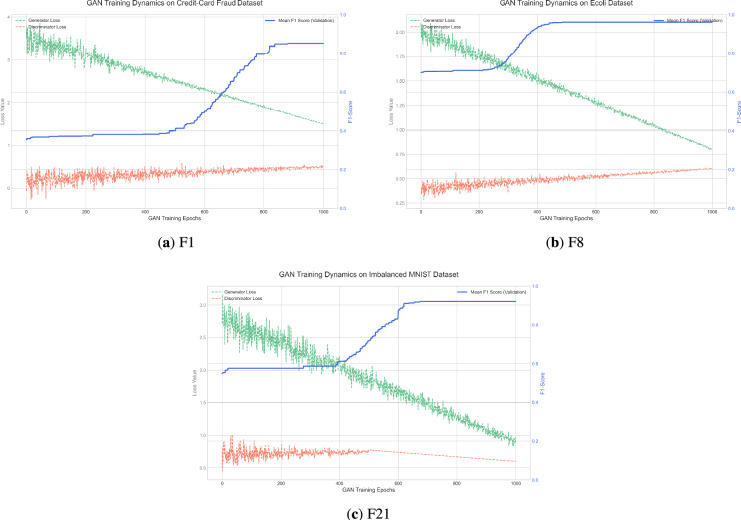
Precision-Recall (PR) curves of different methods on the Credit-Card Fraud dataset.

To further analyze the results from the perspectives of process and effect, we have visualized the optimization process and classifier performance. Figure 5 reveals the internal dynamics of the GAN model trained with the optimal hyperparameters found by E-IVYA. The figure shows that the loss curves of the generator and discriminator eventually stabilize during their adversarial game, without collapsing. At the same time, the F1-Score on the validation set steadily increases and converges to a high level. This provides indirect evidence that the hyperparameter combination found by E-IVYA not only leads to excellent final results but also ensures a healthy and efficient GAN training process. Figure 6 displays the Precision-Recall (PR) curves for each method on the most challenging Credit-Card Fraud dataset. The PR curve for E-IVYA is closest to the top-right corner of the plot and achieves the highest AUC-PR value (0.88). This is strong evidence of superior classifier performance in imbalanced classification evaluation. It indicates that the model enhanced by the E-IVYA-optimized GAN can achieve a higher recall rate than other methods while maintaining high precision.

In summary, the E-IVYA-GAN framework demonstrates exceptional performance, whether viewed from the final quantitative performance metrics (Table 3, Fig. 4), the efficiency and stability of the optimization process (Fig. 5), or the in-depth evaluation of classifier performance (Fig. 6). The experimental results prove that E-IVYA, as an advanced optimizer, has great potential for solving complex and computationally expensive AI problems like GAN hyperparameter optimization.

**Table 3:** Comparison of classification performance of each optimisation method on different datasets.

Dataset	Evaluation metric	No augmentation	SMOTE	GAN (Random Search)	GAN (PSO)	GAN (GWO)	GAN (Original IVYA)	GAN (JADE)	GAN (BO)	GAN (E-IVYA)
Credit-Card Fraud	F1-Score	0.56 (0.05)	0.71 (0.04)	0.77 (0.05)	0.80 (0.03)	0.81 (0.03)	0.82 (0.02)	0.83 (0.02)	0.84 (0.02)	0.87 (0.01)
	G-Mean	0.68 (0.04)	0.81 (0.03)	0.84 (0.04)	0.87 (0.02)	0.87 (0.02)	0.88 (0.02)	0.89 (0.01)	0.89 (0.02)	0.91 (0.01)
	AUC-PR	0.59 (0.06)	0.73 (0.03)	0.79 (0.04)	0.82 (0.03)	0.82 (0.03)	0.83 (0.02)	0.84 (0.02)	0.85 (0.01)	0.88 (0.01)
Ecoli	F1-Score	0.69 (0.06)	0.81 (0.04)	0.84 (0.04)	0.86 (0.03)	0.86 (0.03)	0.87 (0.02)	0.88 (0.02)	0.88 (0.02)	0.91 (0.01)
	G-Mean	0.75 (0.05)	0.86 (0.03)	0.88 (0.03)	0.90 (0.02)	0.90 (0.02)	0.91 (0.02)	0.92 (0.01)	0.92 (0.01)	0.94 (0.01)
	AUC-PR	0.71 (0.05)	0.83 (0.04)	0.86 (0.03)	0.88 (0.03)	0.88 (0.02)	0.89 (0.02)	0.90 (0.02)	0.90 (0.01)	0.92 (0.01)
Imbalanced MNIST	F1-Score	0.85 (0.03)	0.90 (0.02)	0.92 (0.02)	0.93 (0.01)	0.93 (0.01)	0.94 (0.01)	0.95 (0.01)	0.95 (0.01)	0.97 (0.01)
	G-Mean	0.89 (0.02)	0.93 (0.01)	0.94 (0.01)	0.95 (0.01)	0.95 (0.01)	0.96 (0.01)	0.96 (0.01)	0.97 (0.01)	0.98 (0.01)
	AUC-PR	0.87 (0.03)	0.92 (0.02)	0.93 (0.02)	0.94 (0.01)	0.94 (0.01)	0.95 (0.01)	0.96 (0.01)	0.96 (0.01)	0.98 (0.01)

## Conclusion and future work

This paper proposed a multi-strategy synergistic Enhanced Ivy Algorithm (E-IVYA) to address common challenges in metaheuristic optimization, such as premature convergence and susceptibility to local optima. Through comprehensive experiments on the IEEE CEC 2014 and 2017 benchmark suites and a complex application involving GAN hyperparameter optimization, the proposed E-IVYA has demonstrated robust and competitive performance in this study. The experimental results indicate that E-IVYA achieves statistically significant improvements over the baseline IVYA and several state-of-the-art competitors on the majority of the tested functions, as supported by the Wilcoxon signed-rank tests.

**Fig. 7:**
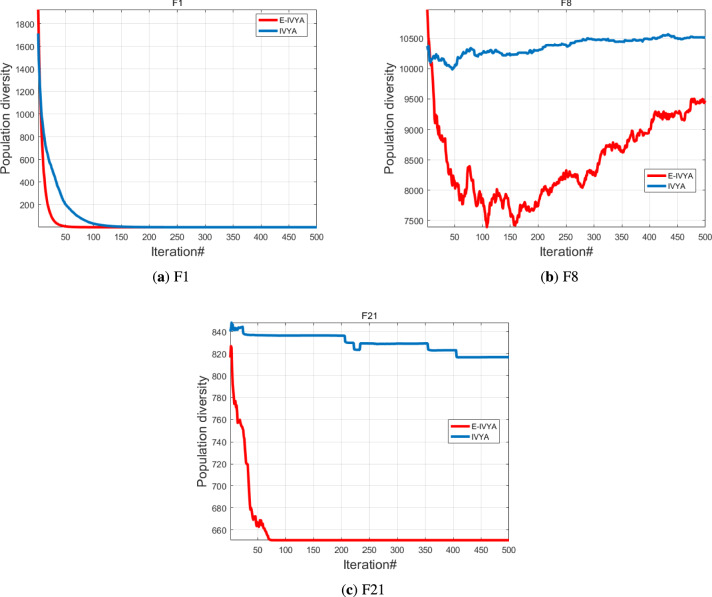
Diversity analysis with the iterations based on three different functions.

**Fig. 8:**
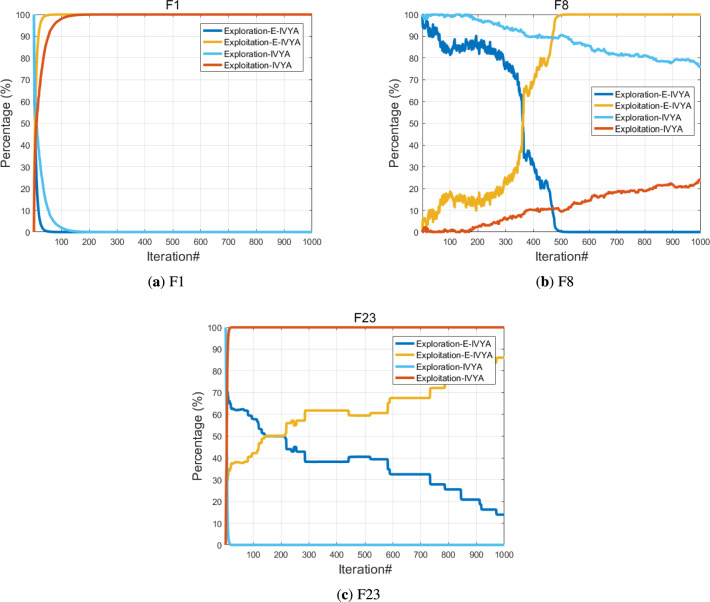
Exploration and exploitation analysis with the iterations based on four different functions.

**Fig. 9:**
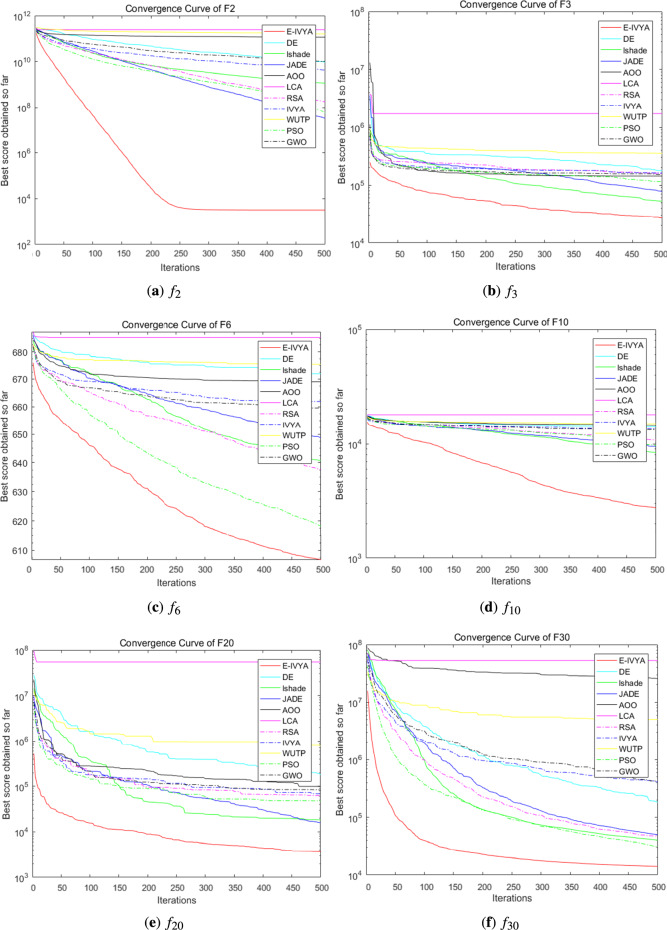
Convergence curves for selected IEEE CEC 2014 benchmark functions (50D). Overall, the plots illustrate that E-IVYA (solid red line) consistently achieves faster convergence rates and superior final solution accuracy on the majority of the test functions, particularly on complex multimodal and composite problems like $f_{21}$ and $f_{30}$.

**Table 4:** The mean values for E-IVYA and other comparative algorithms on IEEE CEC 2014 benchmark functions (50D).

Function	E-IVYA	IVYA	PSO	GWO	DE	RSA	JADE	LSHADE	AOO	LCA	WUTP
F1	**1.8157e+06**	3.5900e+08	1.0116e+09	4.6111e+08	4.3986e+08	1.3781e+07	2.4456e+06	1.9749e+06	9.2227e+09	2.1389e+09	1.7725e+07
F2	**3.1021e+03**	1.9051e+08	9.4005e+10	6.7820e+06	3.4682e+08	2.2289e+04	3.8400e+03	1.7651e+07	2.1197e+11	1.1398e+11	1.1925e+09
F3	**1.5835e+04**	1.2829e+05	1.2001e+05	6.4022e+04	8.2173e+04	1.1578e+05	2.6687e+04	2.9567e+04	7.0428e+05	2.9238e+05	1.1963e+05
F4	**4.7950e+02**	1.0114e+03	2.0553e+04	5.1093e+02	5.7339e+02	5.2537e+02	4.8023e+02	5.2323e+02	1.7513e+05	2.8989e+04	1.3418e+03
F5	5.2346e+02	5.2471e+02	5.3529e+02	5.3400e+02	5.2718e+02	5.3475e+02	5.3562e+02	**5.1769e+02**	5.3216e+02	5.2255e+02	5.2562e+02
F6	**5.9136e+02**	6.5772e+02	6.8290e+02	6.2084e+02	6.8048e+02	6.3533e+02	6.5599e+02	6.2570e+02	6.8996e+02	6.8488e+02	6.6436e+02
F7	**6.8617e+02**	7.1478e+02	1.6231e+03	6.9463e+02	7.2340e+02	7.1517e+02	7.0397e+02	6.9320e+02	3.2504e+03	1.8384e+03	7.2185e+02
F8	**8.4357e+02**	1.2144e+03	1.4057e+03	8.9321e+02	1.2585e+03	8.9856e+02	9.4759e+02	8.6575e+02	1.5869e+03	1.5126e+03	1.2785e+03
F9	1.1356e+03	1.3853e+03	1.5744e+03	1.1594e+03	1.3968e+03	**1.0776e+03**	1.1539e+03	1.1294e+03	1.9329e+03	1.6853e+03	1.4326e+03
F10	**1.4429e+03**	1.2529e+04	1.4481e+04	4.5459e+03	1.2847e+04	5.5682e+03	5.3789e+03	3.2796e+03	1.8465e+04	1.4654e+04	1.2818e+04
F11	**9.9016e+03**	1.4889e+04	1.5317e+04	1.4616e+04	1.5732e+04	1.4618e+04	1.1311e+04	1.0560e+04	1.8546e+04	1.5034e+04	1.4439e+04
F12	1.2335e+03	1.2325e+03	1.2189e+03	1.2303e+03	1.2461e+03	1.2307e+03	1.2215e+03	**1.1852e+03**	1.2324e+03	1.2361e+03	1.2104e+03
F13	**1.2941e+03**	1.3325e+03	1.3343e+03	1.3090e+03	1.3195e+03	1.3370e+03	1.3323e+03	1.3259e+03	1.3217e+03	1.3268e+03	1.3312e+03
F14	**1.3781e+03**	1.4420e+03	1.6384e+03	1.4363e+03	1.4131e+03	1.4394e+03	1.4339e+03	1.4118e+03	2.1190e+03	1.7481e+03	1.4323e+03
F15	**1.4996e+03**	1.8902e+03	5.1559e+05	1.5583e+03	1.6569e+03	1.5540e+03	1.5593e+03	1.5901e+03	6.9458e+07	5.5683e+06	4.2372e+03
F16	1.6521e+03	1.6319e+03	1.6644e+03	1.6499e+03	1.6631e+03	1.6477e+03	1.6607e+03	**1.6116e+03**	1.6524e+03	1.6635e+03	1.6528e+03
F17	**1.6881e+05**	1.8596e+07	1.7554e+08	1.8633e+06	1.4395e+07	2.0837e+06	2.3025e+05	1.7455e+05	1.6562e+09	1.4705e+08	1.6247e+07
F18	**2.1559e+03**	2.5029e+03	9.2968e+09	2.8550e+03	1.9567e+05	2.5539e+03	2.2155e+03	5.7601e+03	2.8727e+10	2.5152e+09	3.4253e+03
F19	**1.8847e+03**	2.0163e+03	2.6288e+03	1.9689e+03	1.9961e+03	1.9620e+03	1.9427e+03	1.9863e+03	5.6883e+03	2.3734e+03	2.0007e+03
F20	**2.8083e+03**	3.5517e+04	7.1197e+04	1.8860e+04	5.9525e+04	4.1030e+04	4.8693e+03	3.1493e+03	2.4599e+06	2.6841e+05	5.0601e+04
F21	1.0506e+05	6.0792e+06	2.3486e+07	1.2869e+06	2.5229e+06	2.3831e+06	**1.0041e+05**	4.5165e+05	5.9189e+08	4.5422e+07	4.8696e+06
F22	**3.2183e+03**	3.9634e+03	8.4116e+03	3.2662e+03	3.9790e+03	3.3283e+03	3.3448e+03	3.9213e+03	3.9797e+06	5.2346e+03	3.9142e+03
F23	2.6508e+03	2.6835e+03	2.9231e+03	2.6559e+03	2.6853e+03	2.7090e+03	2.6628e+03	**2.6071e+03**	5.4674e+03	3.4443e+03	2.7157e+03
F24	2.6937e+03	2.6732e+03	2.7262e+03	2.6358e+03	2.7270e+03	2.6838e+03	2.7489e+03	**2.6042e+03**	3.1936e+03	2.8712e+03	**2.6042e+03**
F25	2.7303e+03	2.7661e+03	2.7788e+03	2.7844e+03	2.7758e+03	2.7839e+03	2.7562e+03	**2.6853e+03**	3.4904e+03	2.9550e+03	2.7865e+03
F26	**2.6687e+03**	2.7719e+03	2.8488e+03	2.8361e+03	2.7618e+03	2.7523e+03	2.7831e+03	2.7735e+03	3.3618e+03	2.7885e+03	2.8362e+03
F27	**3.0123e+03**	4.3146e+03	5.3094e+03	3.3444e+03	4.5453e+03	3.4616e+03	3.7937e+03	4.0264e+03	5.5186e+03	5.0345e+03	4.3411e+03
F28	**4.1729e+03**	5.9329e+03	1.4468e+04	4.2952e+03	4.4140e+03	4.3400e+03	5.1718e+03	4.9818e+03	1.6373e+04	9.5855e+03	6.3986e+03
F29	**4.5670e+03**	1.0560e+06	1.4055e+09	6.6496e+03	6.3275e+04	4.7932e+03	5.6190e+03	6.7083e+07	8.3242e+08	2.9463e+08	1.7667e+06
F30	**1.1783e+04**	1.3530e+05	2.6105e+07	1.9567e+04	2.4578e+04	1.9680e+04	3.2530e+04	2.6186e+04	4.2929e+07	4.2796e+06	1.9288e+05
**Rank 1**	**18**	0	0	0	0	1	1	6	0	0	4

Boldface indicates the algorithm that obtained the optimal experimental result for a particular test function in this test suite.

**Table 5:** The mean values for E-IVYA and other comparative algorithms on IEEE CEC 2014 benchmark functions (100D).

Function	E-IVYA	IVYA	PSO	GWO	DE	RSA	JADE	LSHADE	AOO	LCA	WUTP
F1	**8.6341e+07**	3.1203e+09	1.4589e+10	1.2587e+08	1.5168e+09	2.2965e+08	1.0373e+08	9.0416e+07	8.3045e+09	1.1578e+10	1.5435e+09
F2	**1.4925e+04**	8.2435e+10	4.3129e+11	2.6502e+08	6.4462e+10	1.9961e+09	3.1118e+04	4.9080e+08	2.9158e+11	4.5630e+11	6.1362e+10
F3	**8.7548e+04**	3.4868e+05	9.2736e+05	2.1867e+05	3.0306e+05	3.0182e+05	1.4913e+05	1.0117e+05	7.2343e+05	7.7946e+05	3.1257e+05
F4	**6.4215e+02**	8.9482e+03	1.5204e+05	8.5750e+02	1.0601e+04	1.0822e+03	6.9427e+02	6.8378e+02	1.7699e+05	1.5701e+05	1.0336e+04
F5	**5.1794e+02**	5.2443e+02	5.2533e+02	5.2541e+02	5.2281e+02	5.2638e+02	5.2434e+02	5.2348e+02	5.2592e+02	5.2283e+02	5.2423e+02
F6	6.8443e+02	7.4042e+02	7.5959e+02	6.8834e+02	7.4219e+02	6.9416e+02	6.7901e+02	**6.6021e+02**	7.6432e+02	7.5796e+02	7.2995e+02
F7	**6.9482e+02**	1.5833e+03	4.5427e+03	7.1824e+02	1.2965e+03	7.1997e+02	7.0396e+02	7.0425e+02	3.6339e+03	5.0645e+03	1.2721e+03
F8	**1.0334e+03**	1.9443e+03	2.1466e+03	1.1718e+03	1.9761e+03	1.1593e+03	1.2185e+03	1.1328e+03	2.2964e+03	2.6713e+03	1.9381e+03
F9	1.6373e+03	2.1585e+03	2.3421e+03	1.8385e+03	2.1472e+03	**1.5034e+03**	1.6433e+03	1.6033e+03	3.0617e+03	3.0626e+03	2.2348e+03
F10	**6.8462e+03**	3.0976e+04	3.2429e+04	1.8109e+04	2.9515e+04	1.5039e+04	1.1666e+04	8.2166e+03	3.3218e+04	3.2798e+04	2.9248e+04
F11	**2.9461e+04**	3.2435e+04	3.3768e+04	3.2201e+04	3.0648e+04	3.1783e+04	2.9868e+04	2.9641e+04	3.4214e+04	3.2753e+04	3.0368e+04
F12	**1.1917e+03**	1.2141e+03	1.2263e+03	1.2104e+03	1.2201e+03	1.2230e+03	1.2111e+03	1.2183e+03	1.2290e+03	1.2102e+03	1.2120e+03
F13	**1.2882e+03**	1.3149e+03	1.3283e+03	1.3094e+03	1.3259e+03	1.3219e+03	1.3090e+03	1.3204e+03	1.3347e+03	1.3323e+03	1.3147e+03
F14	**1.3916e+03**	1.6669e+03	2.3023e+03	1.4116e+03	1.5976e+03	1.4159e+03	1.4246e+03	1.4217e+03	2.2575e+03	2.7214e+03	1.5847e+03
F15	**1.5312e+03**	1.2384e+06	1.6573e+07	1.6360e+03	4.9080e+05	2.0006e+03	1.6685e+03	1.5746e+03	7.0267e+07	2.1465e+08	4.8519e+05
F16	**1.6318e+03**	1.6601e+03	1.6558e+03	1.6616e+03	1.6675e+03	1.6521e+03	1.6508e+03	1.6499e+03	1.6582e+03	1.6622e+03	1.6631e+03
F17	3.8447e+06	2.8576e+08	1.1873e+09	1.7062e+07	1.7145e+08	3.9317e+07	4.2257e+06	**3.7088e+06**	1.4328e+09	1.1691e+09	1.6934e+08
F18	**2.2854e+03**	3.1518e+03	3.5510e+10	6.0142e+04	4.0913e+04	3.0336e+03	2.5036e+03	2.4344e+03	2.8188e+10	2.5938e+10	4.0734e+04
F19	**1.9806e+03**	2.3429e+03	8.6834e+03	2.0112e+03	2.2274e+03	2.0005e+03	2.0359e+03	2.0559e+03	6.0717e+03	6.0645e+03	2.2612e+03
F20	**3.5601e+04**	3.0827e+05	7.0988e+05	8.4116e+04	2.5857e+05	2.0396e+05	5.1481e+04	4.1485e+04	2.4549e+06	3.2530e+06	2.5367e+05
F21	**2.6502e+06**	1.0560e+08	3.0617e+08	7.4249e+06	6.2644e+07	7.3751e+06	3.1970e+06	2.9554e+06	5.8624e+08	4.7891e+08	6.3197e+07
F22	**3.9789e+03**	6.8361e+03	4.0673e+04	5.1764e+03	6.8530e+03	7.0863e+03	4.8115e+03	4.5422e+03	3.9317e+06	9.9754e+03	7.0253e+03
F23	2.7171e+03	2.9258e+03	6.0640e+03	2.8465e+03	2.9620e+03	**2.6241e+03**	2.7533e+03	2.6750e+03	5.3789e+03	5.9231e+03	2.9224e+03
F24	2.8366e+03	3.1257e+03	4.0488e+03	2.8023e+03	**2.5973e+03**	2.8596e+03	2.7523e+03	2.7483e+03	3.1965e+03	3.9317e+03	3.0903e+03
F25	2.8252e+03	3.1278e+03	3.6300e+03	2.8106e+03	**2.6844e+03**	2.7915e+03	2.7744e+03	2.7237e+03	3.4478e+03	3.8329e+03	3.1365e+03
F26	**2.7142e+03**	3.0950e+03	3.1200e+03	2.8385e+03	2.8016e+03	2.8597e+03	2.8223e+03	2.8344e+03	3.3283e+03	2.7667e+03	2.8389e+03
F27	**3.9213e+03**	6.6496e+03	8.6946e+03	4.5369e+03	6.2847e+03	5.1233e+03	4.6300e+03	4.2981e+03	5.5970e+03	7.4975e+03	6.4673e+03
F28	**6.3533e+03**	1.9567e+04	2.3556e+04	7.1519e+03	2.2154e+04	1.2016e+04	8.2323e+03	7.4836e+03	1.6253e+04	2.3060e+04	2.2570e+04
F29	**6.7121e+03**	1.6372e+08	1.8386e+09	1.3039e+05	4.9603e+07	1.8492e+04	8.7909e+04	1.2828e+05	8.6019e+08	1.4927e+09	4.9123e+07
F30	**3.6942e+04**	7.3787e+06	8.8471e+07	1.5034e+05	3.2505e+06	1.1449e+05	9.9079e+04	8.0805e+04	4.2562e+07	6.5516e+07	3.2505e+06
Rank 1	**24**	0	0	0	2	2	0	2	0	0	0

Boldface indicates the algorithm that obtained the optimal experimental result for a particular test function in this test suite.

**Table 6:** The mean values for E-IVYA and other comparative algorithms on IEEE CEC 2017 benchmark functions (50D).

Function	E-IVYA	IVYA	PSO	GWO	DE	RSA	JADE	LSHADE	AOO	LCA	WUTP
F1	**1.1396e+06**	2.1481e+09	7.0298e+09	3.0132e+08	3.7337e+08	2.7686e+09	4.0722e+06	1.3522e+07	1.0315e+08	5.1843e+06	4.1039e+06
F2	**2.7698e+03**	9.7186e+10	2.4507e+11	4.5492e+07	3.1678e+08	1.4588e+11	6.6416e+03	5.5901e+07	1.6214e+11	5.7611e+03	6.2082e+08
F3	**9.6417e+02**	1.3283e+05	1.7610e+05	1.3481e+05	1.6841e+05	1.5583e+05	1.5888e+04	1.6419e+04	1.6318e+05	9.8784e+03	1.5791e+04
F4	**4.8812e+02**	2.2173e+04	5.2154e+04	3.2359e+03	8.3286e+02	3.5186e+04	5.3180e+02	6.1387e+02	3.4975e+04	5.0345e+02	5.3262e+02
F5	**5.1742e+02**	5.2494e+02	5.2505e+02	5.2185e+02	5.2514e+02	5.2493e+02	5.2346e+02	5.1837e+02	5.2536e+02	5.2562e+02	5.2325e+02
F6	6.2798e+02	6.6908e+02	6.7580e+02	6.4719e+02	6.6119e+02	6.7865e+02	6.3094e+02	**6.0822e+02**	6.6787e+02	6.2423e+02	6.3683e+02
F7	**6.9416e+02**	1.5971e+03	2.2344e+03	9.1128e+02	7.0863e+02	2.0628e+03	7.0427e+02	7.0451e+02	2.2882e+03	7.0232e+02	7.0465e+02
F8	**8.4983e+02**	1.3481e+03	1.4168e+03	1.0597e+03	1.2059e+03	1.4729e+03	9.1328e+02	8.6826e+02	1.4449e+03	8.7997e+02	9.2155e+02
F9	1.1278e+03	1.4923e+03	1.6253e+03	1.2078e+03	1.3725e+03	1.6508e+03	1.1542e+03	**1.0927e+03**	1.5861e+03	1.1271e+03	1.1667e+03
F10	**3.3854e+03**	1.4172e+04	1.5540e+04	8.6475e+03	1.3204e+04	1.4394e+04	5.6179e+03	3.7915e+03	1.5369e+04	3.6335e+03	5.6457e+03
F11	**4.8368e+03**	1.4589e+04	1.5969e+04	1.2177e+04	1.3854e+04	1.5539e+04	1.1895e+04	1.0335e+04	1.5831e+04	9.8009e+03	1.1965e+04
F12	**1.1925e+03**	1.2069e+03	1.2136e+03	1.2033e+03	1.2069e+03	1.2078e+03	1.2062e+03	1.2093e+03	1.2111e+03	1.2092e+03	1.2087e+03
F13	**1.2882e+03**	1.3168e+03	1.3134e+03	1.3094e+03	1.3087e+03	1.3174e+03	1.3081e+03	1.3090e+03	1.3193e+03	1.3094e+03	1.3072e+03
F14	**1.3888e+03**	1.6429e+03	1.8021e+03	1.4674e+03	1.4103e+03	1.7061e+03	1.4057e+03	1.4116e+03	1.7854e+03	1.4093e+03	1.4042e+03
F15	**1.5008e+03**	4.8159e+05	5.9189e+06	3.5350e+04	1.5796e+03	3.5701e+06	1.5459e+03	1.5721e+03	5.9620e+06	1.5303e+03	1.5398e+03
F16	**1.6107e+03**	1.6263e+03	1.6293e+03	1.6315e+03	1.6247e+03	1.6288e+03	1.6290e+03	1.6253e+03	1.6258e+03	1.6277e+03	1.6290e+03
F17	**4.1209e+04**	2.2858e+08	8.4119e+08	2.6710e+07	8.4239e+06	5.0934e+08	2.9248e+05	3.7371e+05	8.5137e+08	2.0264e+05	2.9463e+05
F18	**2.7681e+03**	7.2144e+09	2.4939e+10	2.9972e+08	4.0202e+05	1.3768e+10	2.7876e+03	3.1932e+03	2.4727e+10	2.9649e+03	2.8091e+03
F19	1.9547e+03	1.9934e+03	2.6393e+03	2.0967e+03	1.9442e+03	4.0150e+03	**1.9168e+03**	1.9708e+03	5.3402e+03	1.9536e+03	1.9599e+03
F20	**2.3888e+03**	8.6385e+04	4.3592e+05	6.5165e+04	4.2980e+04	3.2201e+05	1.4593e+04	1.2163e+04	4.4143e+05	4.2255e+03	1.4552e+04
F21	8.5447e+04	1.8322e+07	1.3664e+08	6.4764e+06	6.1952e+06	9.0116e+07	**8.4189e+04**	2.6366e+05	1.3789e+08	8.9740e+04	8.5443e+04
F22	**2.6953e+03**	1.1738e+04	8.0494e+05	3.5855e+03	4.2505e+03	1.6961e+05	3.4410e+03	3.6146e+03	8.1130e+05	3.3932e+03	3.4475e+03
F23	2.6644e+03	2.9555e+03	2.5186e+03	2.8550e+03	2.6517e+03	2.5097e+03	**2.6101e+03**	2.6622e+03	2.5109e+03	2.6288e+03	2.6580e+03
F24	2.6968e+03	2.6738e+03	2.6166e+03	2.6841e+03	2.7154e+03	2.6144e+03	2.6941e+03	2.7132e+03	2.6166e+03	2.6946e+03	**2.5761e+03**
F25	**2.6741e+03**	2.7235e+03	2.7145e+03	2.7214e+03	2.7562e+03	2.7161e+03	2.7483e+03	2.7454e+03	2.7118e+03	2.7303e+03	2.7431e+03
F26	**2.7458e+03**	2.8080e+03	2.8020e+03	2.7845e+03	2.8122e+03	2.8105e+03	2.7938e+03	2.7865e+03	2.8084e+03	2.7820e+03	2.7946e+03
F27	**3.2843e+03**	5.3411e+03	5.1091e+03	4.1565e+03	4.3972e+03	4.9818e+03	3.5901e+03	3.8443e+03	5.1118e+03	3.5960e+03	3.5976e+03
F28	**3.5761e+03**	1.5599e+04	1.2844e+04	3.6334e+03	6.5840e+03	1.1738e+04	5.1278e+03	5.3134e+03	1.2981e+04	4.5824e+03	5.1215e+03
F29	**3.0768e+03**	1.3090e+09	3.1241e+03	3.1539e+03	4.0305e+07	2.4646e+07	7.3912e+03	1.3023e+07	3.1205e+03	6.7088e+06	7.3794e+03
F30	**3.9984e+03**	1.8331e+07	4.5583e+06	4.0722e+03	1.0768e+05	5.1979e+06	2.1158e+04	2.2343e+04	4.5529e+06	1.4162e+04	2.1121e+04
Rank 1	**22**	0	0	0	0	0	2	3	0	0	3

Boldface indicates the algorithm that obtained the optimal experimental result for a particular test function in this test suite.

**Table 7:** The mean values for E-IVYA and other comparative algorithms on IEEE CEC 2017 benchmark functions (100D).

Function	E-IVYA	IVYA	PSO	GWO	DE	RSA	JADE	LSHADE	AOO	LCA	WUTP
F1	**1.4392e+07**	4.0436e+09	1.0853e+10	9.3242e+08	2.6888e+09	8.1678e+09	6.5501e+07	1.6961e+08	1.1077e+10	6.0954e+07	6.6415e+07
F2	**1.5794e+04**	2.1624e+11	3.1234e+11	5.5413e+09	1.2584e+10	2.8687e+11	9.8038e+07	1.9213e+07	3.1554e+11	1.9568e+07	1.0366e+10
F3	**1.6778e+04**	2.8643e+05	3.1787e+05	3.2982e+05	4.2934e+05	3.1493e+05	8.0776e+04	9.8887e+04	3.1818e+05	9.9241e+04	8.1994e+04
F4	**7.4011e+02**	5.3341e+04	1.1189e+05	1.1368e+04	2.3087e+03	7.8937e+04	8.6836e+02	1.5123e+03	1.1147e+05	7.6322e+02	8.6946e+02
F5	**5.1783e+02**	5.2343e+02	5.2477e+02	5.2536e+02	5.2560e+02	5.2435e+02	5.2415e+02	5.2329e+02	5.2285e+02	5.2522e+02	5.2598e+02
F6	6.9248e+02	7.5583e+02	7.6041e+02	7.1352e+02	7.5928e+02	7.6250e+02	7.0263e+02	**6.4589e+02**	7.6166e+02	6.7410e+02	7.0427e+02
F7	**6.9531e+02**	2.9099e+03	3.8443e+03	1.6258e+03	8.0441e+02	3.5513e+03	7.0673e+02	7.5746e+02	3.8903e+03	7.0543e+02	7.0617e+02
F8	**1.0123e+03**	2.0318e+03	2.1934e+03	1.5898e+03	1.8105e+03	2.2530e+03	1.3687e+03	1.2464e+03	2.1741e+03	1.3697e+03	1.3695e+03
F9	1.6888e+03	2.2741e+03	2.4042e+03	1.7018e+03	1.9798e+03	2.3831e+03	1.7291e+03	**1.6083e+03**	2.4013e+03	1.7299e+03	1.7311e+03
F10	**1.1417e+04**	3.1365e+04	3.3283e+04	2.1332e+04	3.0569e+04	3.1265e+04	1.8519e+04	1.3533e+04	3.3312e+04	1.7088e+04	1.8317e+04
F11	**1.0718e+04**	3.0678e+04	3.2687e+04	2.5742e+04	3.1866e+04	3.1189e+04	2.7845e+04	2.5649e+04	3.2694e+04	2.5202e+04	2.7876e+04
F12	**1.1920e+03**	1.2069e+03	1.2063e+03	1.2075e+03	1.2071e+03	1.2043e+03	1.2065e+03	1.2060e+03	1.2057e+03	1.2091e+03	1.2053e+03
F13	**1.2911e+03**	1.3168e+03	1.3175e+03	1.3150e+03	1.3056e+03	1.3160e+03	1.3061e+03	1.3055e+03	1.3161e+03	1.3060e+03	1.3041e+03
F14	**1.3912e+03**	2.0537e+03	2.3423e+03	1.6436e+03	1.4385e+03	2.2351e+03	1.4079e+03	1.4191e+03	2.3444e+03	1.4082e+03	1.4073e+03
F15	**1.5401e+03**	4.7679e+06	3.0270e+07	2.5936e+05	4.3582e+04	1.4842e+07	1.6429e+03	2.7758e+03	3.0288e+07	1.6130e+03	1.6593e+03
F16	**1.6318e+03**	1.6570e+03	1.6582e+03	1.6599e+03	1.6572e+03	1.6568e+03	1.6464e+03	1.6457e+03	1.6575e+03	1.6577e+03	1.6467e+03
F17	**8.1001e+05**	7.4764e+08	2.0718e+09	1.0460e+08	8.4485e+07	1.2152e+09	3.7126e+06	6.6669e+06	2.0729e+09	4.0321e+06	3.7135e+06
F18	**2.6314e+03**	2.1834e+10	4.7208e+10	2.3551e+09	6.3073e+06	3.5113e+10	3.8211e+03	4.6190e+05	4.7196e+10	4.2371e+03	3.8239e+03
F19	**1.9968e+03**	5.8569e+03	1.3078e+04	2.6154e+03	2.1724e+03	9.0700e+03	2.0526e+03	2.0917e+03	1.3082e+04	2.0398e+03	2.0504e+03
F20	**3.5711e+03**	4.2562e+05	1.3283e+06	2.9157e+05	7.1528e+05	8.3644e+05	6.7621e+04	7.1329e+04	1.3287e+06	5.2583e+04	6.7601e+04
F21	1.5592e+06	1.8010e+08	6.2974e+08	5.3743e+07	3.6705e+07	3.7732e+08	**1.4988e+06**	3.2592e+06	6.2996e+08	1.7408e+06	1.5191e+06
F22	**3.3012e+03**	1.8412e+04	4.6678e+05	5.6987e+03	7.5097e+03	1.0560e+05	5.5539e+03	5.8827e+03	4.6685e+05	5.4729e+03	5.5524e+03
F23	2.6953e+03	3.1594e+03	2.5159e+03	3.2687e+03	2.7369e+03	2.5153e+03	2.6659e+03	2.7274e+03	2.5165e+03	2.6664e+03	**2.4811e+03**
F24	2.6634e+03	2.7663e+03	2.6171e+03	2.6158e+03	2.9378e+03	2.6166e+03	2.8465e+03	2.9080e+03	2.6158e+03	2.8471e+03	**2.5619e+03**
F25	**2.6804e+03**	2.7461e+03	2.7163e+03	2.7570e+03	2.8778e+03	2.7153e+03	2.8166e+03	2.8152e+03	2.7158e+03	2.7997e+03	2.8152e+03
F26	**2.7788e+03**	2.8158e+03	2.8173e+03	2.8272e+03	2.8772e+03	2.8167e+03	2.8160e+03	2.8159e+03	2.8167e+03	2.8165e+03	2.8163e+03
F27	**4.0199e+03**	8.3512e+03	8.0898e+03	5.9149e+03	5.8239e+03	7.3829e+03	4.7865e+03	5.5830e+03	8.0886e+03	4.6860e+03	4.7877e+03
F28	**5.2711e+03**	3.6318e+04	2.9377e+04	5.4121e+03	1.4485e+04	1.9616e+04	1.3175e+04	1.0152e+04	2.9379e+04	6.9582e+03	1.3179e+04
F29	**3.0784e+03**	2.0125e+09	3.1189e+03	3.1593e+03	3.4812e+07	3.1190e+03	1.1666e+04	2.1121e+07	3.1189e+03	1.7505e+04	1.1670e+04
F30	**6.4021e+03**	1.4285e+08	1.0456e+07	6.6908e+03	8.3653e+05	1.0760e+07	1.6896e+05	1.4891e+05	1.0457e+07	2.8763e+04	1.6904e+05
Rank 1	**25**	0	0	0	0	1	1	1	0	0	2

Boldface indicates the algorithm that obtained the optimal experimental result for a particular test function in this test suite.

The primary contributions of this work are fourfold. First, we introduced a dynamic perturbation framework combining elite-guided symmetric exploration and adaptive asymmetric perturbation to effectively maintain population diversity. Second, we designed an intelligent escape mechanism based on elite differential mutation, providing the algorithm with a guided capability to escape from local optima. Third, an adaptive movement strategy integrating the principles of the Sine-Cosine Algorithm (SCA) was developed for an improved dynamic balance between exploration and exploitation. Finally, the application of the E-IVYA-GAN framework *demonstrates the potential capability* of the proposed algorithm in solving computationally expensive, real-world AI optimization tasks.

**Fig. 10:**
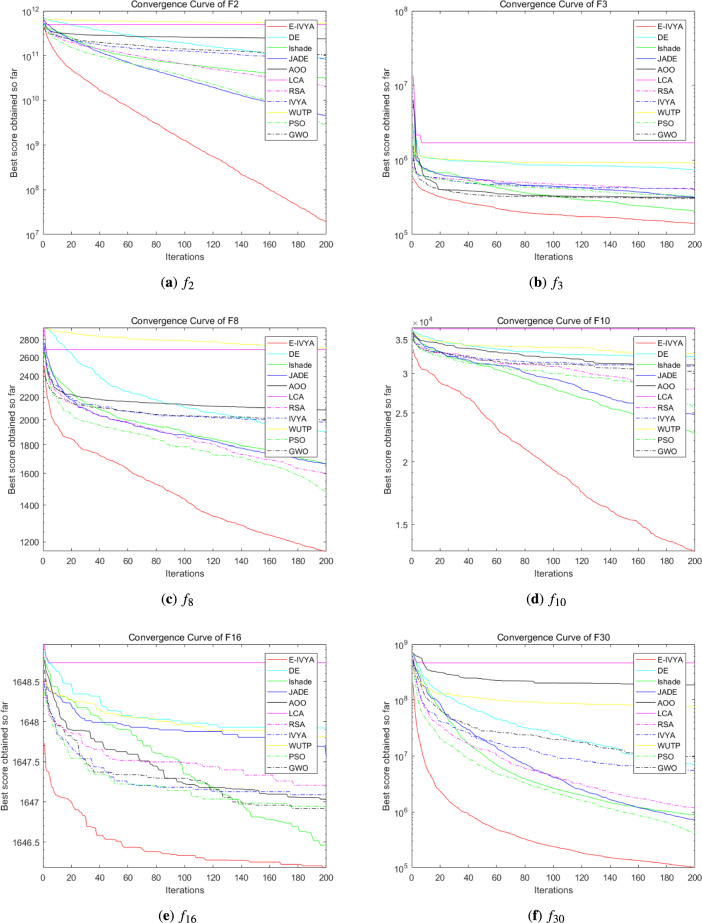
Convergence curves for selected IEEE CEC 2014 benchmark functions (100D). In the higher-dimensional setting, the performance advantage of E-IVYA becomes more pronounced, demonstrating its strong scalability and robustness by maintaining rapid convergence and high precision across different function types.

**Fig. 11:**
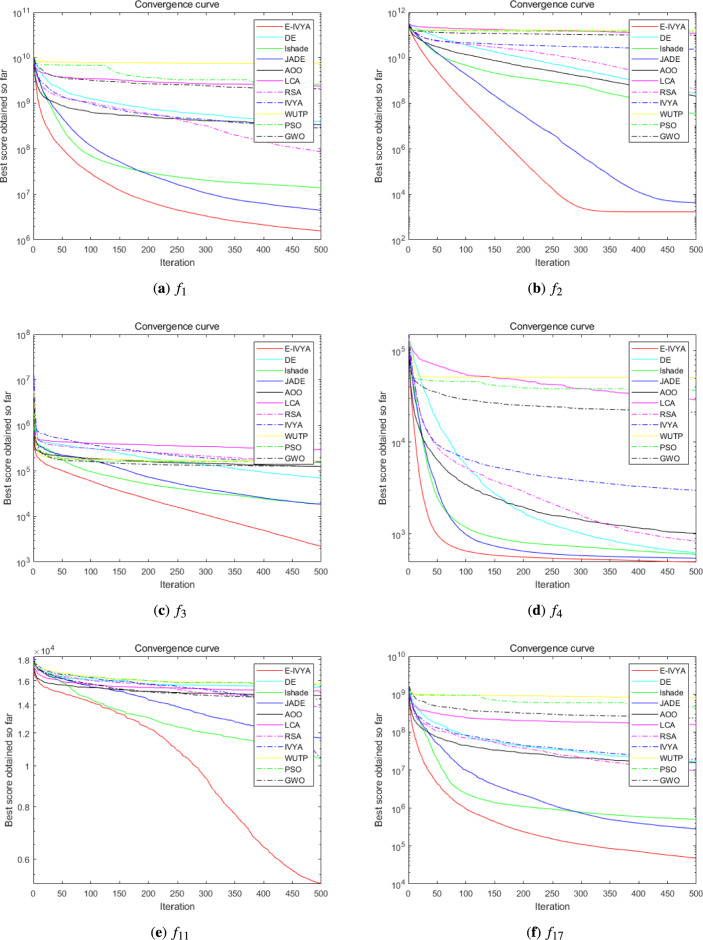
Convergence curves for selected IEEE CEC 2017 benchmark functions (50D). On this more challenging test suite, E-IVYA continues to exhibit its superiority, outperforming advanced DE variants like JADE and LSHADE on most of the presented functions, which highlights the effectiveness of its synergistic enhancement strategies.

**Fig. 12:**
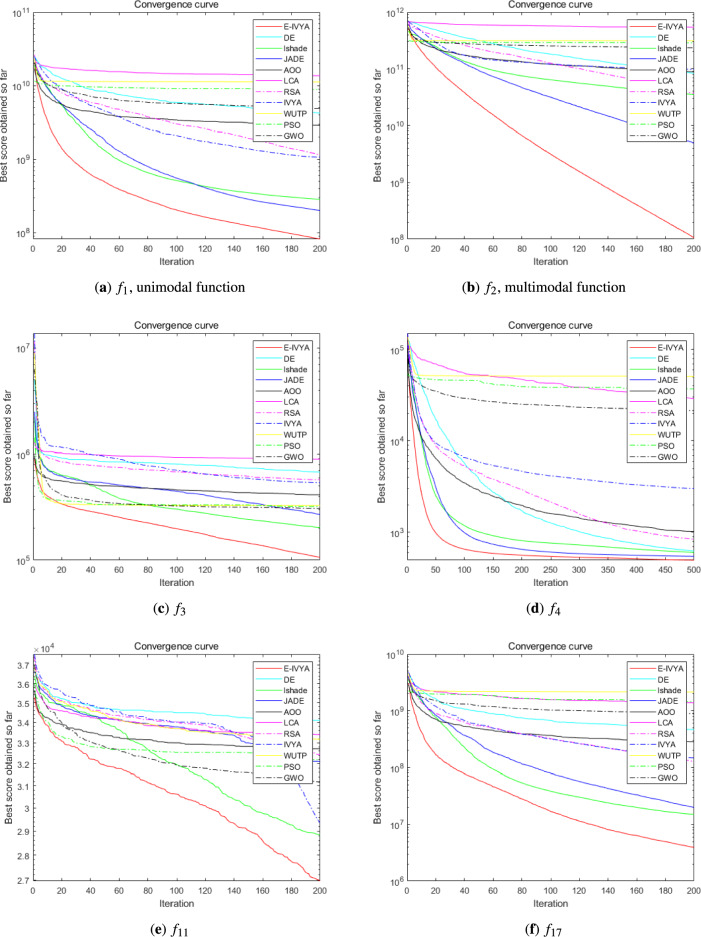
Convergence curves for selected IEEE CEC 2017 benchmark functions (100D). The results at 100 dimensions further confirm E-IVYA’s robust performance and scalability, as it consistently finds better solutions than its competitors in a highly complex and high-dimensional search space.

**Fig. 13:**
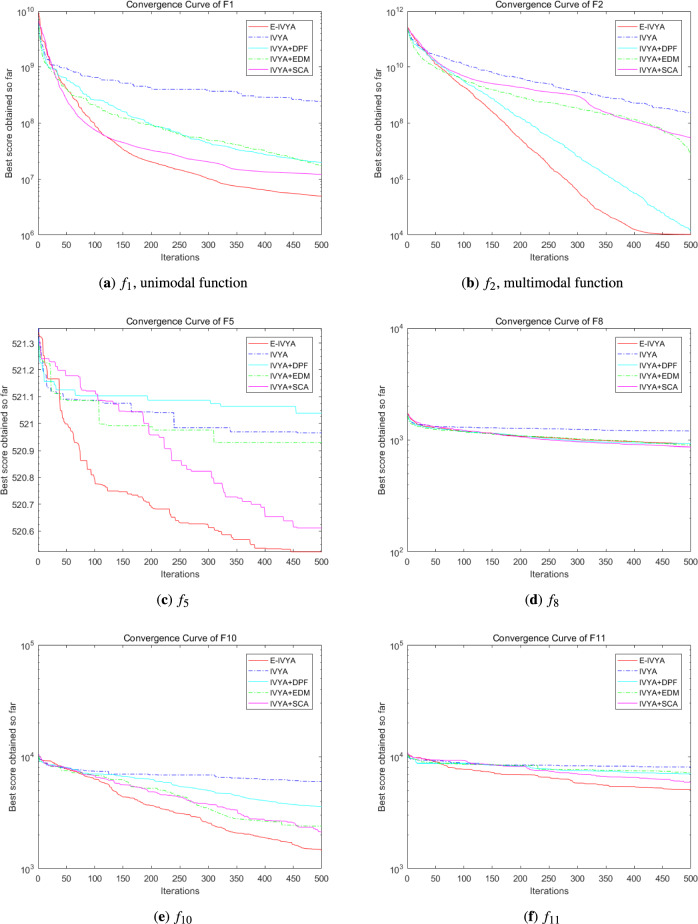
Ablation Study of E-IVYA Components.

**Fig. 14:**
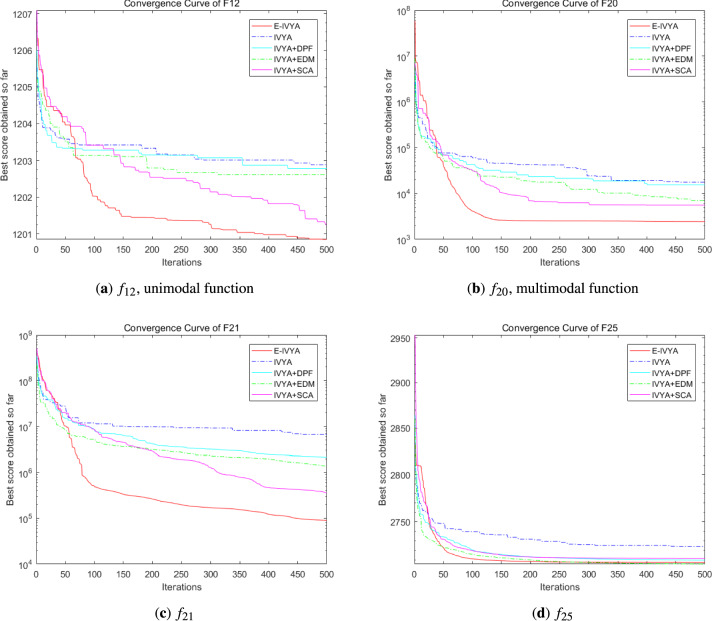
Ablation Study of E-IVYA Components.

**Fig. 15:**
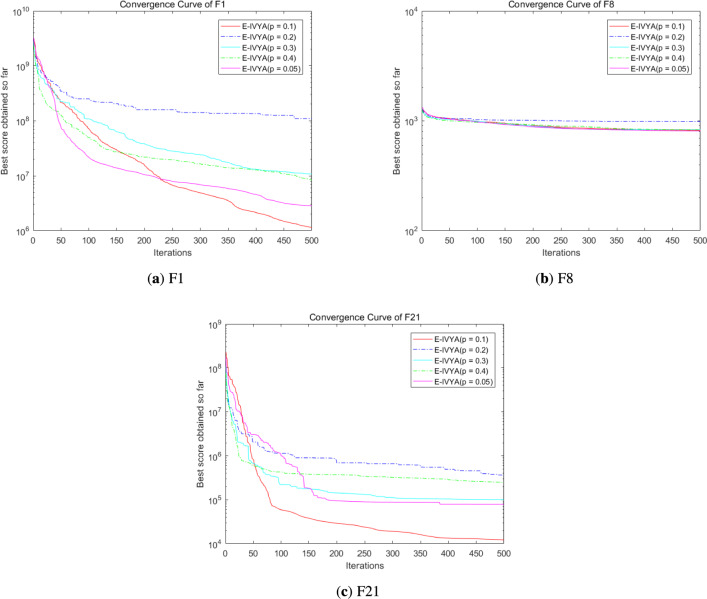
Sensitivity analysis of the elite archive percentage (*p*), with *F* fixed at 0.5.

**Fig. 16:**
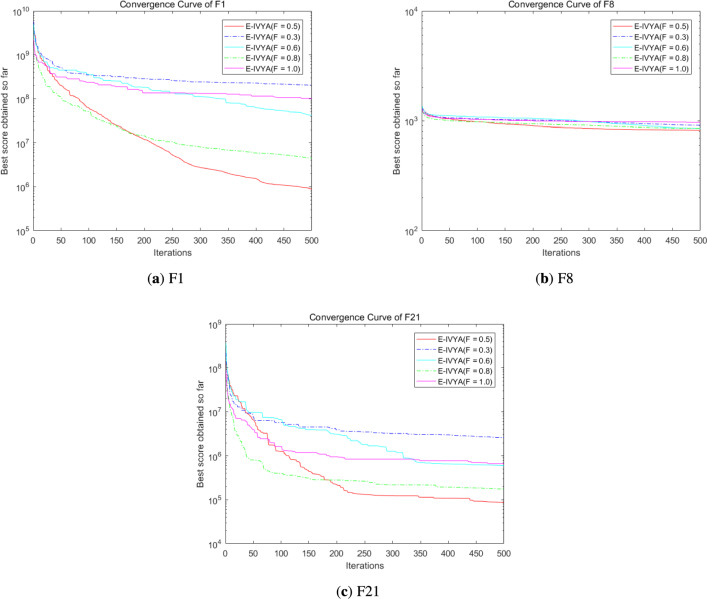
Sensitivity analysis of the scaling factor (*F*), with *p* fixed at 0.1.

Despite its promising performance, E-IVYA has several limitations that warrant discussion. From an algorithmic perspective, the integration of new mechanisms increases its structural complexity and introduces control parameters that currently lack self-adaptivity. More importantly, as detailed in our runtime analysis, these enhancements lead to a higher computational cost per iteration (approximately 1.26x to 1.37x) compared to the original IVYA. This trade-off between improved optimization accuracy and increased runtime cost is a critical consideration, as the higher overhead might make E-IVYA less suitable for applications with extremely tight time constraints. From a problem-modeling perspective, the E-IVYA-GAN framework simplifies the complex task of GAN evaluation into a single-objective optimization problem. This approach, while effective in this study, may overlook other critical quality aspects such as sample fidelity and intra-class diversity. Finally, while validated on several datasets, the generalizability of E-IVYA to even higher-dimensional problems or other generative models remains an open question requiring further investigation.

**Table 8:** Results of the Wilcoxon’s signed-rank test for E-IVYA and other meta-heuristic algorithms on IEEE CEC 2014 benchmark functions.

E-IVYA vs	IVYA	PSO	GWO	DE	RSA	JADE	LSHADE	AOO	LCA	WUTP
*50D*										
*p*	1.15E-06	1.73E-06	1.73E-06	1.73E-06	1.36E-05	4.86E-05	3.15E-03	1.24E-05	1.60E-04	1.73E-06
$\alpha = 0.1$	Yes	Yes	Yes	Yes	Yes	Yes	Yes	Yes	Yes	Yes
$\alpha = 0.05$	Yes	Yes	Yes	Yes	Yes	Yes	Yes	Yes	Yes	Yes
*100D*										
*p*	1.13E-06	1.73E-06	1.73E-06	1.73E-06	1.13E-05	6.16E-05	8.42E-02	7.16E-05	1.24E-04	1.73E-06
$\alpha = 0.1$	Yes	Yes	Yes	Yes	Yes	Yes	Yes	Yes	Yes	Yes
$\alpha = 0.05$	Yes	Yes	Yes	Yes	Yes	Yes	No	Yes	Yes	Yes

**Table 9:** Results of the Wilcoxon’s signed-rank test for E-IVYA and other meta-heuristic algorithms on IEEE CEC 2017 benchmark functions.

E-IVYA vs	IVYA	PSO	GWO	DE	RSA	JADE	LSHADE	AOO	LCA	WUTP
*50D*										
*p*	1.24E-05	1.73E-06	1.99E-05	4.86E-05	1.73E-06	9.32E-04	4.11E-02	9.32E-05	2.38E-04	1.60E-05
$\alpha = 0.1$	Yes	Yes	Yes	Yes	Yes	Yes	Yes	Yes	Yes	Yes
$\alpha = 0.05$	Yes	Yes	Yes	Yes	Yes	Yes	Yes	Yes	Yes	Yes
*100D*										
*p*	1.13E-05	1.73E-06	1.13E-05	6.16E-05	1.73E-06	1.24E-03	9.58E-02	1.24E-04	7.16E-04	4.86E-05
$\alpha = 0.1$	Yes	Yes	Yes	Yes	Yes	Yes	Yes	Yes	Yes	Yes
$\alpha = 0.05$	Yes	Yes	Yes	Yes	Yes	Yes	No	Yes	Yes	Yes

**Table 10:** Performance Comparison of IVYA Variants.

Test platform	Metric	IVYA (Original)	IVYA + DPF (Imp. 1)	IVYA + EDM (Imp. 2)	IVYA + SCA (Imp. 3)	E-IVYA (Complete)
CEC 2014 F1 (50D)	Mean Fitness	$2.44 \times 10^8$	$1.98 \times 10^7$	$1.70 \times 10^7$	$1.84 \times 10^7$	$3.35 \times 10^6$
CEC 2014 F8 (50D)	Mean Fitness	$1.19 \times 10^3$	$9.23 \times 10^2$	$8.92 \times 10^2$	$8.64 \times 10^2$	$8.2678 \times 10^2$
CEC 2014 F21 (50D)	Mean Fitness	$6.66 \times 10^6$	$2.15 \times 10^6$	$1.37 \times 10^6$	$1.27 \times 10^5$	$1.12 \times 10^5$
Credit-Card Fraud	F1-Score	0.82 (0.02)	0.83 (0.02)	0.85 (0.02)	0.84 (0.02)	0.87 (0.01)

**Table 11:** Empirical Runtime Analysis (Average Wall-Clock Time per Run)

Test platform	Metric	IVYA (Baseline)	PSO (Classic)	JADE (Advanced)	E-IVYA (Ours)
CEC 2014 F1 (50D)	Mean Time (s)	62.51	51.33	71.20	85.43
	Norm. Cost	1.00x	0.82x	1.14x	1.37x
CEC 2014 F8 (50D)	Mean Time (s)	65.84	53.12	74.91	90.17
	Norm. Cost	1.00x	0.81x	1.14x	1.37x
CEC 2014 F21 (50D)	Mean Time (s)	81.33	68.70	92.42	110.61
	Norm. Cost	1.00x	0.84x	1.14x	1.36x
GAN Optimization	Mean Time (h)	8.17	7.92	9.06	10.31
(Credit-Card)	Norm. Cost	1.00x	0.97x	1.11x	1.26x

Based on the findings and limitations of this study, several promising directions for future research are apparent. To address parameter sensitivity, future work could focus on designing self-adaptive mechanisms, potentially using reinforcement learning, to dynamically adjust control parameters during the run. To tackle the single-objective limitation, extending E-IVYA into a multi-objective optimization framework is a compelling direction. For instance, integrating E-IVYA with a well-established multi-objective framework could allow for the simultaneous optimization of both classification performance and sample diversity metrics. Finally, the optimization capabilities of E-IVYA could be applied to a wider range of complex AI tasks, such as Neural Architecture Search (NAS) or policy optimization in reinforcement learning.

## Data Availability

The source code for the proposed E-IVYA algorithm is publicly available on GitHub at https://github.com/jack66668888/E-IVYA. The datasets used in this study, including the Credit-Card Fraud dataset, the Ecoli dataset, and the constructed Imbalanced MNIST dataset, are publicly archived on Zenodo at https://doi.org/10.5281/zenodo.17424661. The IEEE CEC 2014 and CEC 2017 benchmark suites are standard, publicly known test functions commonly used in the field.
